# Functional conservation of HIV-1 Gag: implications for rational drug design

**DOI:** 10.1186/1742-4690-10-126

**Published:** 2013-10-31

**Authors:** Guangdi Li, Jens Verheyen, Soo-Yon Rhee, Arnout Voet, Anne-Mieke Vandamme, Kristof Theys

**Affiliations:** 1 Rega Institute for Medical Research, Department of Microbiology and Immunology, KU Leuven, Leuven, Belgium; 2 Institute of Virology, University hospital, University Duisburg-Essen, Essen, Germany; 3 Division of Infectious Diseases, Department of Medicine, Stanford University, Stanford, CA, USA; 4 Zhang IRU, RIKEN Institute Laboratories, Hirosawa 2-1, Wako-shi, Saitama, Japan; 5 Centro de Malária e Outras Doenças Tropicais and Unidade de Microbiologia, Instituto de Higiene e Medicina Tropical, Universidade Nova de Lisboa, Lisbon, Portugal

**Keywords:** HIV subtype, Gag inhibitor, Matrix, Capsid, Nucleocapsid, Drug binding site, Natural polymorphism, Amino acid conservation

## Abstract

**Background:**

HIV-1 replication can be successfully blocked by targeting gag gene products, offering a promising strategy for new drug classes that complement current HIV-1 treatment options. However, naturally occurring polymorphisms at drug binding sites can severely compromise HIV-1 susceptibility to gag inhibitors in clinical and experimental studies. Therefore, a comprehensive understanding of gag natural diversity is needed.

**Findings:**

We analyzed the degree of functional conservation in 10862 full-length gag sequences across 8 major HIV-1 subtypes and identified the impact of natural variation on known drug binding positions targeted by more than 20 gag inhibitors published to date. Complete conservation across all subtypes was detected in 147 (29%) out of 500 gag positions, with the highest level of conservation observed in capsid protein. Almost half (41%) of the 136 known drug binding positions were completely conserved, but all inhibitors were confronted with naturally occurring polymorphisms in their binding sites, some of which correlated with HIV-1 subtype. Integration of sequence and structural information revealed one drug binding pocket with minimal genetic variability, which is situated at the N-terminal domain of the capsid protein.

**Conclusions:**

This first large-scale analysis of full-length HIV-1 gag provided a detailed mapping of natural diversity across major subtypes and highlighted the considerable variation in current drug binding sites. Our results contribute to the optimization of gag inhibitors in rational drug design, given that drug binding sites should ideally be conserved across all HIV-1 subtypes.

## Introduction

A curative therapy or preventive vaccine for HIV-1 infected patients remains elusive to date. Standard HIV treatment is confronted with the emergence of viral resistance to existing drug classes, necessitating the development of inhibitors with new mechanisms of action [1]. The gag polyprotein, essential for HIV-1 morphogenesis, comprises four major domains (matrix, capsid, nucleocapsid, p6) and two small spacer peptides (p1, p2) [2]. Recently, HIV-1 inhibitors that target different stages of virion morphogenesis demonstrated promising antiviral activity, mainly by inhibiting capsid assembly, disrupting nucleocapsid binding with viral RNA/DNA or blocking proteolytic processing of polyproteins during maturation [2-5].

HIV-1 subtype B isolates were predominantly used for the *in vitro* experiments. Non-B subtypes however account for 90% of HIV-1 infections worldwide [6] and amino acid (AA) compositions can differ up to 30% between subtypes [7]. Recently, treatment failure of patients in a phase II clinical study of the maturation inhibitor bevirimat was attributed to natural polymorphisms at drug binding positions, showing up in subtype-specific patterns [8]. Studies that extensively investigate the implications of HIV-1 diversity for gag-directed drug development are lacking to date. In this large-scale analysis, we examined the distribution of naturally occurring sequence variability in full-length gag sequences of major HIV-1 subtypes. Moreover, we evaluated the impact of HIV-1 subtypes on the conservation of gag drug binding positions and multisite binding pockets published to date.

## Results

We analyzed 10862 full-length gag sequences that fulfilled the quality criteria, encompassing 8 HIV-1 group M subtypes and CRFs: A1 (n = 1648), B (n = 4131), C (n = 2780), D (n = 443), F1 (n = 35), G (n = 49), CRF01_AE (n = 1714) and CRF02_AG (n = 62). Sequences were sampled from 61 countries between 1981 and 2012. Additional file 1: Table S1 summarizes more than 50 gag inhibitors including their binding sites, target protein, mechanism of action, HIV-1 subtypes and PDB data. These candidate inhibitors were either small organic molecules or peptides and primarily targeted the capsid or nucleocapsid proteins. A total of 136 gag positions were reported as drug binding positions, of which 53 interacted with more than one inhibitor.

**Figure 1:**
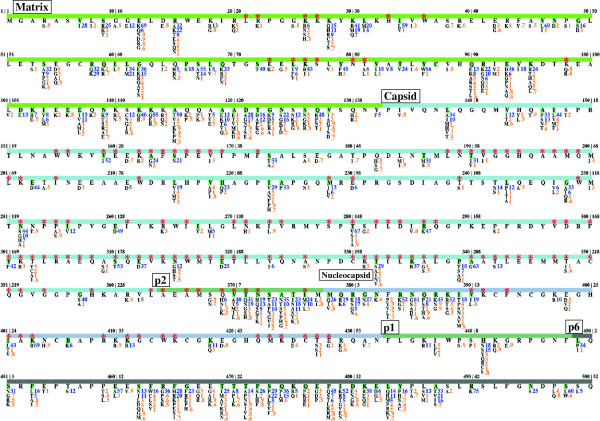
**Distribution of natural variations at 500 gag positions of HIV-1 group M (subtypes: A1, B, C, D, F1, G and CRF01_AE, CRF02_AG).** The first position of each protein region is labeled with its protein name in a box. Annotated protein regions are indicated as colored bars: light-green for matrix (positions 1–132), light-blue for capsid (133–363), dark-green for p2 (364–377) and p1 (433–448), dark-blue for nucleocapsid (378–432) and grey for p6 (449–500). HXB2 indices for both full-length gag and individual proteins are shown on top of the colored bars (e.g. '180|48’ indicates the gag position 180 and the capsid position 48). Known drug binding positions are marked with red stars. Consensus subtype B amino acid for each position is shown directly under the bar, and is highlighted green when the consensus AA differed in one or more subtypes. Natural polymorphisms are shown below the consensus subtype B amino acids; proportions (%) are colored blue for proportion ≥ 5%; orange otherwise. Figure S1 in Additional file 2 provides the distribution of natural polymorphisms within each individual subtype.

**Figure 2:**
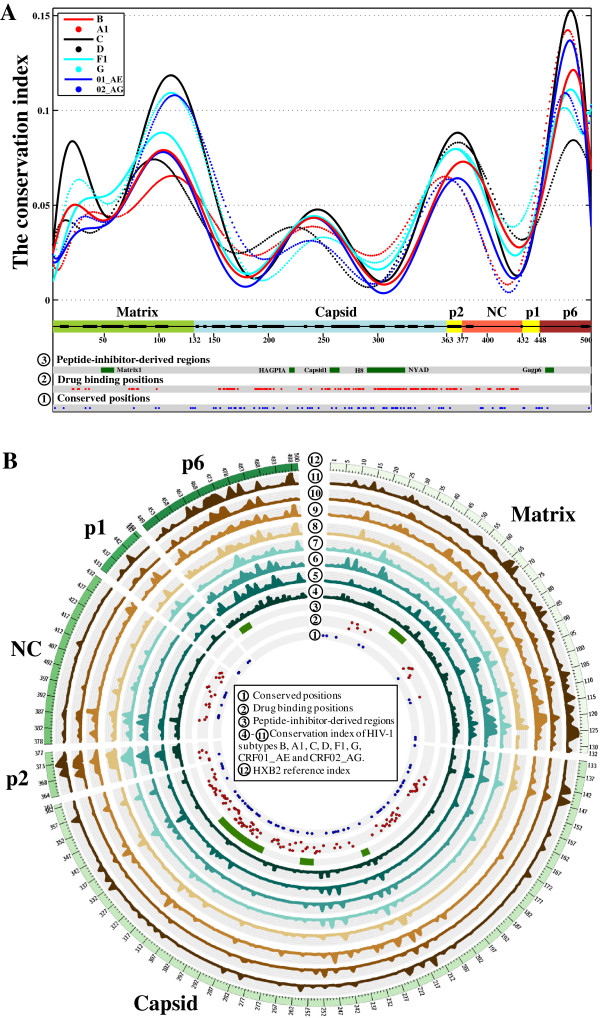
**Amino acid conservation in HIV-1 full-length gag. (A)** Density plots of CI values are shown for 8 HIV-1 subtypes. Secondary structures are indicated for each protein region, with thick lines for helices and thin lines for coiled-coil structures. Positions conserved in all subtypes are colored blue (layer 1 in a small circle), known drug binding positions are colored red (layer 2) and regions where HIV-1 peptide inhibitors have been derived are colored green (layer 3). **(B)** Distributions of CI values at 500 gag positions across 8 HIV-1 subtypes and CRFs. Visualization software: Circos v0.64 (http://circos.ca/).

The AA distribution at 500 gag positions among HIV-1 group M sequences is shown in Figure 1 and subtype-specific distributions are also visualized (Additional file 2: Figure S1). Heterogeneity in consensus sequences was observed at 142 (28.4%) positions across subtypes, while pairwise comparisons of consensus sequences showed an average of 11.6% difference between subtypes. On average, 43.6 ± 2.7% of positions harbored at least one polymorphism relative to its subtype consensus residue (Table 1). The capsid protein (29.4%) contained the lowest number of polymorphic positions followed by nucleocapsid (42.5%), matrix (59.9%), and p6 (65.6%). Moreover, of 147 conserved positions in gag, 67.8% were in capsid, 11.2% in nucleocapsid, 10.5% in matrix and 4.6% in p6. Pairwise AA diversity (Additional file 3) of full-length gag sequences decreased from 17.0 ± 1.6% between subtypes to 9.0 ± 1.0% within subtypes (Table 2). The mean AA diversity was significantly lower for capsid (5.0 ± 0.8%) than for nucleocapsid (7.9 ± 2.8%), matrix (13.2 ± 2.0%) or p6 (14.7 ± 2.0%) (p-value < 0.05) (Table 3). The CI distributions of full-length gag characterized three conserved regions located at the nucleocapsid zinc-finger domains, the capsid N-terminal domain (NTD) and C-terminal domain (CTD) (Figure 2).

**Table 1:** Natural polymorphism proportions in gag domains and drug binding positions across 8 HIV-1 subtypes and CRFs (%)

	**B**	**A1**	**C**	**D**	**F1**	**G**	**01_AE**	**02_AG**	**Mean**
Matrix[132/13]	57.6/38.5	62.1/46.2	59.1/46.2	64.4/53.8	52.3/46.2	66.7/61.5	61.4/46.2	56.1/30.8	59.9/46.2
Capsid[231/98]	27.3/30.6	34.2/33.7	29.4/29.6	27.7/27.6	31.2/37.8	30.3/28.6	28.1/28.6	27.3/27.6	29.4/30.5
p2[14/8]	71.4/62.5	64.3/62.5	64.3/62.5	64.3/62.5	57.1/62.5	71.4/62.5	64.3/62.5	50.0/50.0	63.4/60.9
NC[55/17]	56.4/58.8	41.8/35.3	38.2/41.2	36.4/29.4	34.5/23.5	54.5/58.8	43.6/35.3	34.5/41.2	42.5/40.4
p1[16/0]	37.5/-	25.0/-	31.2/-	43.8/-	25.0/-	31.2/-	31.2/-	12.5/-	29.7/-
p6[52/0]	76.9/-	69.2/-	69.2/-	55.8/-	65.4/-	61.5/-	69.2/-	57.7/-	65.6/-
Mean	45.2/36.8	46.6/36.8	43.4/34.6	42.8/32.4	41.2/38.2	47.0/37.5	44.0/33.1	39.0/30.9	43.6/35.0

Each gag protein is demonstrated with its total AA and drug binding positions (e.g. Matrix [132/13]: 132 AA positions/13 drug binding positions). Natural polymorphism proportion (%) is indicated for each protein and subtype with respect to the total number of AAs and to the number of drug binding positions (e.g. 57.6/38.5 shows that for subtype B matrix, 57.6% of all 132 positions and 38.5% of 13 drug binding positions are polymorphic). Mean values across gag domains and across HIV-1 subtypes are indicated in the last row and column respectively.

**Table 2:** The inter- and intra-subtype diversity of gag AA sequences in 8 HIV-1 subtypes and CRFs (%)

	**Subtype**	**B**	**A1**	**C**	**D**	**F1**	**G**	**01_AE**	**02_AG**
Intra-subtype		8.96	8.34	9.89	8.91	9.45	10.90	7.58	8.26
Inter-subtype	B		17.54	18.38	12.70	16.52	18.46	17.22	18.61
	A1			17.65	16.73	16.93	17.27	12.71	14.69
	C				16.67	17.21	18.02	18.22	19.59
	D					16.55	17.56	16.85	18.72
	F1						15.93	16.48	17.68
	G							17.70	18.81
	01_AE								14.92

**Table 3:** The pairwise AA diversity of gag domains in 8 HIV-1 subtypes and CRFs (%)

	**Matrix**	**Capsid**	**p2**	**NC**	**p1**	**p6**	**Gag**
B	12.36	4.56	20.65	10.31	4.77	15.74	8.84
A1	10.69	5.46	13.97	4.75	5.60	17.17	8.18
C	14.77	5.77	23.79	9.79	4.80	15.83	9.96
D	12.74	4.87	25.62	9.43	8.89	10.17	8.68
F1	12.71	5.37	29.24	9.00	6.71	15.32	9.32
G	17.51	6.15	16.06	10.53	8.42	13.90	10.71
01_AE	11.36	3.44	24.36	4.95	7.74	14.07	7.46
02_AG	13.67	4.91	18.50	3.31	5.39	14.39	8.35
Mean	13.17±2.01	5.04±0.79	21.49±4.83	7.92±2.77	6.39±1.60	14.70±1.99	9.02±1.09

**Figure 3:**
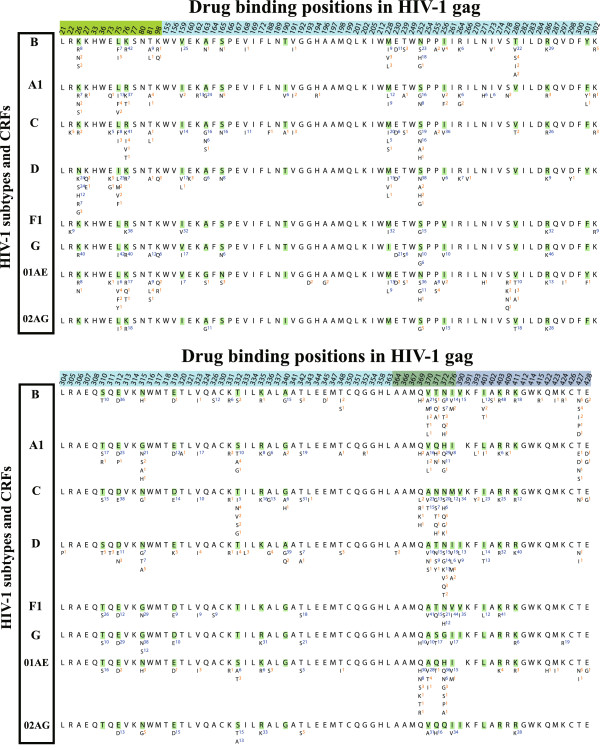
**Natural polymorphisms at 136 drug binding positions in 8 HIV-1 subtypes and CRFs.** For each gag position, the HXB2 index is shown at the top, followed by the consensus amino acid and natural polymorphisms. Polymorphisms with proportions ≥ 5% are indicated with blue superscripts; orange otherwise.

Subtype-specific AA prevalence at the 136 drug binding positions is shown in Figure 3. Most positions were located within capsid (72.1%) followed by nucleocapsid (12.5%), matrix (9.6%) and p2 (5.9%). Of these positions, 41.2% were conserved across all subtypes, while 20.6% showed a different consensus AA in one or more subtypes. On average, 33.8% of drug binding positions harbored at least one polymorphism and 16.3% had at least one polymorphism above 5% prevalence. Non-B subtypes displayed 32 polymorphisms at 20 binding positions that were absent in subtype B. Every inhibitor had at least one polymorphic binding position and 15 inhibitors had more than 50% of drug binding positions showing natural polymorphisms. Among all inhibitors, PF-3450074 [9] targeted the most conserved binding positions at the capsid N-terminal domain, with only one being polymorphic (T107A/S, ≤ 6.2%) (Additional file 1: Table S2).

**Figure 4:**
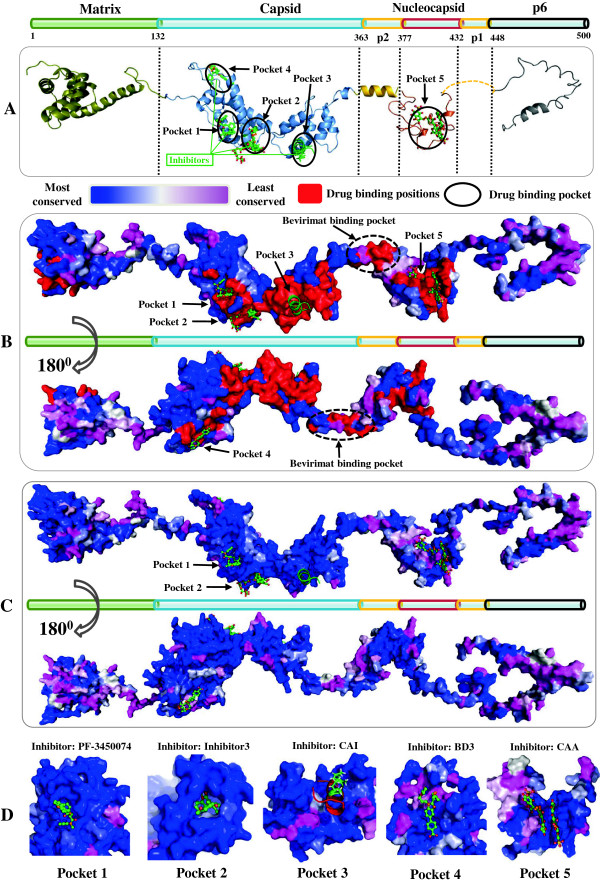
**Mapping of drug binding positions and binding pockets to HIV-1 gag protein monomers.** The surface spectrum colors indicate the most to the least conserved positions in subtype B from blue CI = 0 to pink CI ≥ 0.1. **(A)** Secondary structures of 4 gag proteins and 2 spacer peptides, annotated with five drug binding pocket locations. Gag proteins in cartoon representation are colored olive for matrix, blue for capsid, yellow for nucleocapsid, grey for p6, gold for p1 and p2. Bound inhibitors are represented in green sticks. **(B)** Mapping of drug binding positions to a surface representation of gag structure, with front and back views. Hypothesized binding positions of bevirimat are also annotated; known drug binding positions are colored red. **(C)** Surface representation of gag conservation in HIV-1 subtype B (Figure S3 in Additional file 2 illustrates other subtypes). **(D)** Surface representations of five drug binding pockets in HIV-1 subtype B (Figure S2 in Additional file 2 shows other subtypes). Inhibitor names are annotated according to publication (Additional file 1: Table S1). PDB entries of gag proteins: matrix, 1HIW; capsid, 3NTE; p2, 1U57; nucleocapsid, 2M3Z; p6, 2C55. PDB data of capsid inhibitors: 2BUO, 2L6E, 2XDE, 4E91, 4E92, 2JPR and 4INB, each of which was superimposed to 3H4E using PDBs of 5 drug binding pockets: pocket 1, 2XDE; pocket 2, 4INB; pocket 3, 2BUO; pocket 4, 4E91; pocket 5, 2M3Z. PyMOL V1.5 (http://www.pymol.org/).

Finally, we analyzed known crystal structures of 9 protein-inhibitor complexes, with 8 inhibitors targeting a total of 75 positions (binding pockets 1–4) in capsid and one targeting 23 positions in nucleocapsid (binding pocket 5) (Figure 4, Additional file 2). Natural polymorphisms with prevalence ≥ 5% were observed in 28 positions of the binding pockets. Conserved positions were observed in 56% of the capsid binding pockets and 43% of the nucleocapsid binding pocket. Pocket 1 (0.0024) had the lowest average CI values compared to pocket 2 (0.008), 3 (0.0216), 4 (0.0337) or 5 (0.0369).

## Discussion and conclusions

To our knowledge, our large-scale analysis provided the first detailed mapping of functional conservation of gag across major HIV-1 subtypes, with implications for the rational design of gag inhibitors. With more than 50 gag inhibitors published to date, targeting virion morphogenesis is considered a potential new drug class for HIV-1 treatment [2]. A clinical proof-of-concept was demonstrated in a phase II clinical trial of the maturation inhibitor bevirimat [10], which blocks proteolytic processing at the capsid-p2 cleavage site [11]. Lack of response was observed in 50% of patients and attributed to naturally occurring polymorphisms in the p2 region [8]. A single polymorphism V370A is sufficient for a 40-fold reduction in bevirimat drug susceptibility [12], with A370 representing the consensus amino acid in several non-B subtypes. Natural diversity was also observed to affect drug effectiveness of other experimental gag inhibitors [13-15]. Polymorphisms T190I, E230D and I256V, for instance, reduced drug susceptibility to the benzodiazepine and benzimidazole compounds [13]. Moreover, known HIV vaccine candidates containing subtype B gag gene in HIV-derived vectors did not show sufficient protective efficacies in several large-scale clinical trials [16]. The high diversity of gag and env genes within and between subtypes can contribute to the challenges of designing a global HIV vaccine neutralizing all HIV-1 subtypes [17]. For the development of HIV vaccine and a potential new drug class targeting virion morphogenesis [2], an assessment of gag functional conservation and polymorphisms at known drug binding positions is warranted.

We found that 23.4% of drug binding positions in the full-length gag showed natural polymorphisms in non-B subtypes which could not be detected in subtype B. More importantly, all gag inhibitors had at least one polymorphic binding position irrespective of subtype. We also found levels of gag intra- and inter-subtype diversity (9.04% and 17.0%) that exceeded diversity estimates of key viral enzymes (< 7% and < 11%) targeted by standard HIV-1 treatment [18]. However, the most conserved gag protein capsid has the same level of intra-subtype diversity as integrase (~5%) [18], favoring it as a conserved drug target.

**Figure 5:**
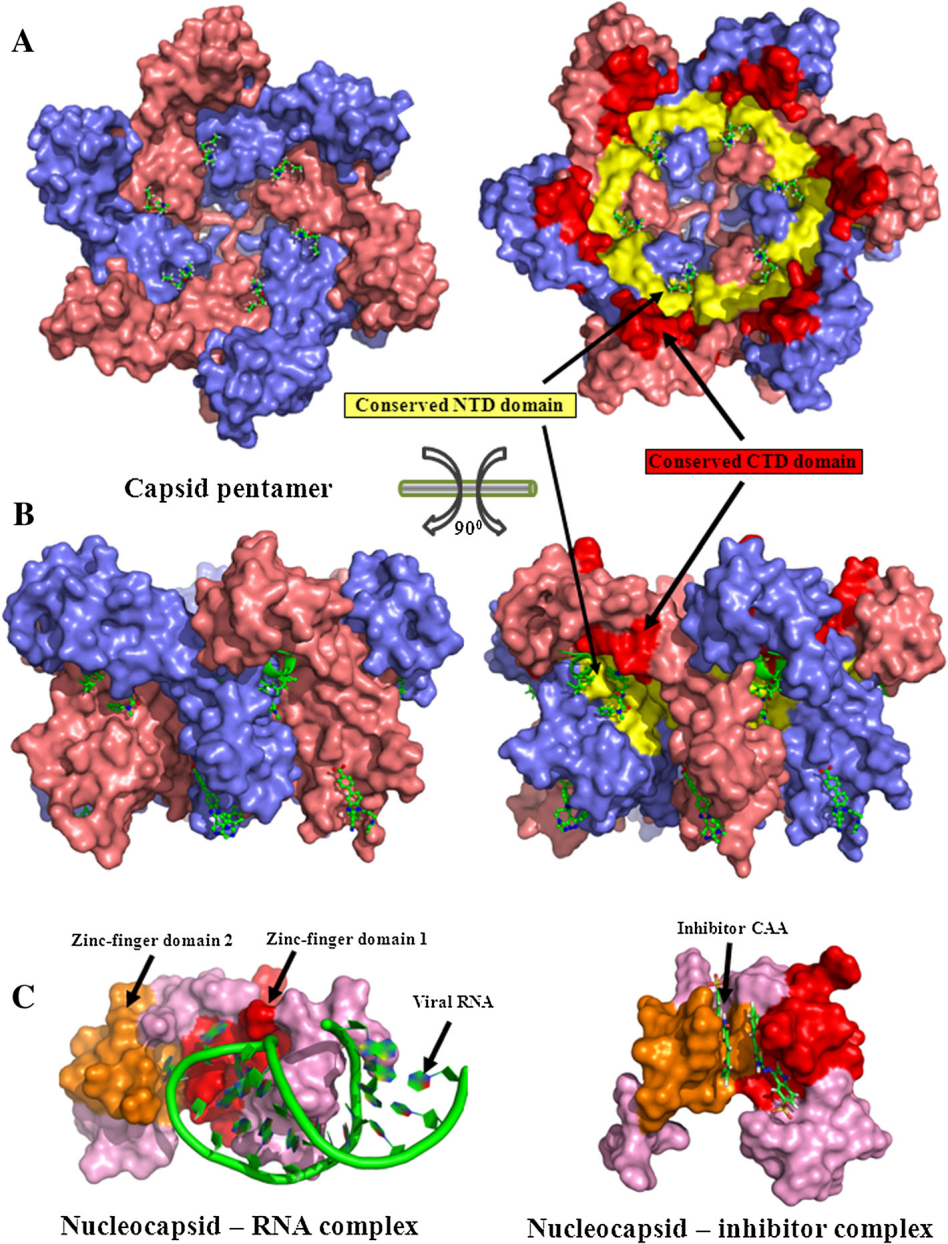
**Visualization of conserved regions in capsid and nucleocapsid.** The capsid hexamer structure (PDB: 3H4E) is shown in top **(A)** and side **(B)** views, with the 6 capsid units (pink, blue), conserved NTD-NTD interaction domains (yellow) and conserved NTD-CTD interaction domains (red). Figure **(C)** shows the structural complex of nucleocapsid and RNA (left, PDB: 1A1T) and the structural complex of nucleocapsid and inhibitor CAA (right, PDB: 2M3Z). The first zinc-finger domain (nucleocapsid positions: 14–29, gag positions: 389–404) and the second zinc-finger domain (nucleocapsid positions: 35–50, gag positions: 410–425) are colored red and orange, respectively. Figures S5 and S6 in Additional file 2 provide detailed structures of conserved gag regions.

The capsid protein targeted by most candidate inhibitors accounted for 67.7% of conserved gag positions and contained 72.1% of the 136 binding positions previously reported. Our sequence analysis identified two conserved capsid regions (Figure 2) located at the interaction interfaces between N-terminal domains (NTD-NTD) as well as between N-terminal and C-terminal domains (NTD-CTD) (Figure 5). These interaction interfaces, crucial for the assembly and stabilization of pentamer and hexamer lattices [19], provide potential conserved drug targets. To reveal the ideal drug target, we described 4 crystalized drug binding pockets in capsid (Figure 4, Additional file 2: Figure S4). Inhibitors that target pockets 1–3 have shown promising antiviral activity against capsid multimerization in different subtype strains by altering NTD-CTD interaction (pockets 1 and 3) or NTD-NTD interaction (pocket 2) [15,20,21]. Pocket 4 is less conserved and its polymorphic residues make direct contact with inhibitors, hindering the development of inhibitors that target this pocket [13].

Another potential drug target is the nucleocapsid protein, containing two critical zinc-finger domains for binding with viral RNA genomes [2]. Our conservation analysis mapped the conserved nucleocapsid regions to zinc-finger domains (Figures 2 and 5) and confirmed previous findings of absolute conservation of CCHC motifs at zinc-coordinating positions [22]. However, we detected considerable variation at other positions, which may alter drug binding and affect antiviral activity. Furthermore, nucleocapsid inhibitors tend to suffer from limited specificity and high toxicity due to the ubiquitous presence of zinc finger domains in many human proteins [4].

Matrix inhibitors with broad spectrum antiviral activities were recently reported, but mutations at drug binding positions significantly reduced their effectiveness [23,24]. We also observed many natural variants at their drug binding sites (Additional file 1: Table S2), suggesting that further optimization of matrix inhibitors is needed.

Studies that analyzed genetic variability and drug binding site heterogeneity in gag using large-scale sequence populations are lacking. Previously, small subtype B sequence datasets were used to characterize gag conservation (n = 125) [25] or positive selective pressure (n = 635) [26]. Polymorphisms at drug binding sites of capsid inhibitor PF-3450074 [9] and conservation of nucleocapsid zinc-finger domains [22] were also reported using fewer than 200 sequences. The only large-scale analysis that we found [27] quantified the drug binding site conservation of a single matrix inhibitor and lacked information on subtype-specific variations. By contrast, we presented here a large-scale and integrative analysis using 10862 full-length gag sequences, 136 gag inhibitor drug binding positions and 14 PDB structures. Natural polymorphisms of full-length gag were detected across 8 major HIV-1 subtypes and a robust estimation of functional conservation was performed using CI analysis, which incorporated biochemical similarities between amino acids (Additional file 3). This sequence analysis predicted three conserved drug targets in gag (Figure 2) which were confirmed by existing structural knowledge (Figure 5).

This study is limited in that it neither addressed how to optimize known gag inhibitors nor quantified the impact of newly identified polymorphisms on antiviral activities of investigated inhibitors. We collected all available PDBs of gag-inhibitor structures from the RCSB protein data bank, but more crystallized complexes are needed to reveal novel mechanisms of action. Moreover, the limited number of available gag sequences for subtypes F1, G and CRF02_AG (n < 100) may have affected the identification of polymorphic positions, but consistent conservation patterns were observed in gag regardless of HIV-1 subtype (Figure 2). While we attempted to be as comprehensive as possible, additional inhibitors may have been reported. Conservation of their binding positions can nevertheless be deduced from our full-length gag analysis. Future studies are also needed to address whether interactions between gag and protease can affect gag drug binding sites, leading to compromised drug activities of gag inhibitors [28].

In conclusion, our study presented a comprehensive mapping of functional conservation in gag and strengthened the idea of capsid as a potential target for HIV-1 therapeutics. Increased knowledge on HIV-1 natural diversity in drug binding pockets contributes to rational design of gag inhibitors and it remains a challenge to design gag inhibitors with drug binding sites conserved across HIV-1 subtypes.

## Methods

We retrieved 12543 gag sequences spanning all 1500 base pairs from the HIV Los Alamos database (http://www.hiv.lanl.gov). Sequences were aligned against the HXB2 reference and manually curated using Seaview 4.3 [29]. Hypermutated sequences were detected using the Los Alamos hypermut tool [30]. HIV-1 subtype was determined by the Rega [31] and COMET subtyping tools (http://comet.retrovirology.lu/). Sequence quality was ensured by excluding duplicates and sequences with internal stop-codons, hypermutations, more than 1% ambiguous nucleotides, discordant subtype classification or an identical combination of patient code, sampling year and country. The analysis was restricted to the major subtypes and circulating recombinant forms (CRFs) characterizing the global HIV-1 subtype distribution [6]. For each individual subtype, amino acids that differed from the corresponding consensus AA and with prevalence ≥ 0.5% were defined as polymorphisms [18]. PDB data of protein-inhibitor complexes were collected from the RCSB Protein Data Bank [32], summarized in Additional file 1. The AA sequences in each PDB were aligned against the HXB2 reference. Drug binding pockets were defined by protein positions within a minimum Euclidean distance of less than 5Å between atoms of inhibitors and non-hydrogen atoms of residues [33]. Information on known gag candidate inhibitors and binding positions was retrieved from more than 50 publications, summarized in Additional file 1.

To quantify the degree of positional conservation, a conservation index (CI) was calculated for each position by averaging pairwise scores between all AAs using the BLOSUM62 substitution matrix. Adapted from Karlin and Brocchieri [34], the conservation index (CI) of position *x* is calculated as: $\mathrm{CI}\left(x\right)=1-\frac{2}{N\left(N-1\right)}\sum_{i=1}^{N}\sum_{j=i+1}^{N}\left[S\left(x_{i},x_{j}\right)/\sqrt{S\left(x_{i},x_{i}\right)S\left(x_{j},x_{j}\right)}\right]$, where *x*_*i*_ is the amino acid at position *x* in the i^th^ sequence of the multiple sequence alignment (MSA), *N* is the number of sequences in the MSA and *S*(*x*_*i*_, *x*_*j*_) is the substitution score of BLOSUM62 between amino acids *x*_*i*_ and *x*_*i*_. Given that denominators cannot be zero, a linear transformation was applied to *S*(*x*_*i*_, *x*_*j*_) by adding the absolute value of the minimum score | min(*S*)| + 1. CI measures were scaled between 0 and 1, with a CI value of 0 indicating that AA variation was absent at that position. A highly conserved position was identified if its CI is below 0.01 for each HIV-1 subtype, a cutoff which corresponds approximately to a cumulative polymorphism prevalence below 1% (Additional file 3). The Mann–Whitney U test was performed to compare CI distributions. Performance of the CI method is evaluated in Additional file 3 and our Matlab toolbox for sequence analysis is available in Additional file 4.

## Abbreviations

AA: amino acid; CI: conservation index; CTD: C-terminal domain; CRF: circulating recombinant form; MSA: multiple sequence alignment; NC: nucleocapsid; NTD: N-terminal domain; PDB: protein data bank.

## Competing interests

The authors declare that they have no competing interests.

## Authors’ contributions

GL and KT conceived and designed the study. All authors have participated in the discussion and interpretation of the results and writing of the manuscript. All authors read and approved the final manuscript.

## Supplementary Material

Additional file 1 **Table S1.** The summary of gag candidate inhibitors published in literature. **Table S2.** The prevalence of natural polymorphisms at known drug binding sites in 8 HIV-1 subtypes and CFRs. Click here for file

Additional file 2 **Figure S1.** The distribution of natural variations of full-length gag in 8 HIV-1 subtypes. **Figure S2.** The surface representation of full-length gag in 8 HIV-1 subtypes and CRFs. **Figure S3.** The surface representation of five drug binding pockets in 8 HIV-1 subtypes. **Figure S3.** The surface representation of full-length gag in 8 HIV-1 subtypes and CRFs. **Figure S4.** The structure of capsid hexamer superimposed with 8 crystalized inhibitors. **Figure S5.** The surface representation of conserved regions in HIV-1 capsid **Figure S6.** The surface representation of conserved regions in HIV-1 nucleocapsid. Click here for file

Additional file 3 **Note S1.** The mathematical model of conservation index. **Note S2.** The mathematical model of inter- and intra-subtype diversity. Click here for file

Additional file 4 **The Matlab toolbox developed for conservation analysis, inter- and intra-subtype diversity analysis.** The full-length gag sequence datasets are also included. Click here for file
