# Supplementary material for: Functional conservation of HIV-1 Gag: implications for rational drug design
Source: Retrovirology. 2013 Oct 31;10:126. doi: 10.1186/1742-4690-10-126 (PMC4228425; doi:10.1186/1742-4690-10-126)
Supplement: Additional file 1 — Table S1. The summary of gag candidate inhibitors published in literature. Table S2. The prevalence of natural polymorphisms at known drug binding sites in 8 HIV-1 subtypes and CFRs. [file 1742-4690-10-126-S1.pdf]

Additional file 1: Tables

Title page

Functional conservation of HIV-1 gag: implications for rational drug design

Authors and Affiliations

Guangdi Li[1], Jens Verheyen[2], Soo-Yon Rhee[1,3], Arnout Voet[4], Anne-Mieke Vandamme[1,5], Kristof Theys[1*]

*Corresponding author: Kristof Theys Kristof.Theys@rega.kuleuven.be

1 Rega Institute, Department of Microbiology and Immunology, KU Leuven, Leuven, Belgium

2 Institute of Virology, University hospital, University Duisburg-Essen, Essen, Germany

3 Division of Infectious Diseases, Department of Medicine, Stanford University, Stanford, California, USA

4 Zhang IRU, RIKEN Institute Laboratories, Hirosawa 2-1, Wako-shi, Saitama, Japan

5 Centro de Malária e Outras Doenças Tropicais and Unidade de Microbiologia, Instituto de Higiene e Medicina Tropical, Universidade Nova de Lisboa, Lisboa, Portugal

Email: Guangdi Li liguangdi.research@gmail.com
Jens Verheyen jens.verheyen@uk-essen.de
Soo-Yon Rhee syrhee@stanford.edu
Arnout Voet arnout.voet@fys.kuleuven.be
Anne-Mieke Vandamme annemie.vandamme@uz.kuleuven.ac.be
Kristof Theys Kristof.Theys@rega.kuleuven.be

Table S1, The summary of experimental gag inhibitors published to date.

	Inhibitor names	Target protein	Binding sites in target protein	Detected effect	HIV strain	PDB	Clinical trial	Year	Ref
**Peptide inhibitors**	Matrix1	Matrix	?	Inhibit assembly and maturation	B			1994	[1]
	Capsid1	Capsid	?	Inhibit assembly and maturation	B			1994	[1]
	CAI	Capsid	D166,Y169,K182,N183,E187,E212,M215	Inhibit capsid CTD interaction	Cell-free	2BUO		2005	[2]
	CAC1/CAC1M,H8	Capsid	I150,R154,Q155,R168,L173,Q179,N184,W185,M186,T189,L191,V192,C199, T201,I202,L203,K204,A205,L212,E213,M215,M216,A218,C219,Q220, V166,L173,E181, N194,D198,K200, G209,A210,T211,V222	Inhibit capsid assembly	B			2011	[3]
	NYAD series	Capsid	V165,F168,Y169,L172,R173,K182,N183,T186,L211,E212,M215[4]	Inhibit capsid CTD interaction	A,B,C,F,G,01AE	2L6E		2011	[5]
	P-1, P-2, P-3, P-4	Capsid	L151,R154, R184, M185, T188, K203(note that only the largest changesof NMR in the presence of peptides were notified)	Inhibit CA polymerization	B			2011	[6]
	CP4	Capsid	T148, I150, L151, D152, V181, A185, T186, Q192, A208, L211, E212, T216	Inhibit CA-hLysRS interaction	Cell-free			2011	[7]
	HAGPIA	CypA	H54,R55,G72,Q63,N102,H126,W121	Inhibit CA-CypA interaction	Cell-free	1AWR		1997	[8]
	Gagp6 (p6:346-354)	TSG101	T58,Y63,R64,Y68,N69,I70,T92,M95,K98,V141,F142,S143	Inhibit TSC101 UEV domain binding with gag	Cell-free	3OBU		2010	[9]
**Small organic molecule**	Compound14	Matrix	?	Inhibit PI(4,5)P$_2$-matrix interaction	A,B,C,D,E,F,G			2012	[10]
	Compound7	Matrix	L21,R22,W36,R76,T81,K98	Inhibit PI(4,5)P$_2$-matrix interaction	A,B,C,D,E,F,G			2013	[11]
	TD1,TD2,TD3	Matrix	L21,R22,K26,K27,H33,W36,E73,L75, R76,S77	Inhibit MA-RNA interaction	B			2013	[12]
	Bevirimat	CA-SP1	?	Inhibit gag maturation	B		Phase IIb[13]	2003	[14]
	Bevirimat analogs (C-28, C-30)	CA-SP1	H358,L363,A364 A366,Q369,A370 T371	Inhibit gag maturation	B			2011	[15]
	Vivecon (MPC-9055)	CA-SP1	?	Inhibit gag maturation	A,B,C,D,E,F,G, group O and N		Phase IIa (discontinued)	2009	[16]
	PA1050040	CA-SP1	?	Inhibit gag maturation	-		Phase I [17]	2009	[18]
	MPI-461359	CA-SP1	?	Inhibit gag maturation	-			2010	[19]
	PF-46396	CA-SP1	?	Inhibit gag maturation	B,C,E[20]			2012	[21]
	Compound(16)	CA-SP1	A366, M367, Q369, V370, N372, I376	Inhibit gag maturation	B			2012	[22]
	I-XW-053	Capsid	S33,P34,E35,V36,V165,D166,F168,Y169,K170,T171,L172,R173,A174,E175, Q176,S178,Q179,E180,N183,T186	Inhibit NTD interaction	A,B,C,D,E,F,G, group O			2012	[23]
	PF-3450074	Capsid	N57,M66,Q67,K70,I73,T107	Inhibit capsid NTD interaction	A,B,C,D,E	2XDE		2010	[24]
	BM4	Capsid	W23,V27A/I,K30R,F32,S33G,T58I,H62	Inhibit capsid NTD interaction	B	4E92		2012	[25]
	BD3	Capsid	W23,V24,V27A/I,F32,V36T,T58I,V59,G61E,H62	Inhibit capsid NTD interaction	B	4E91		2012	[25]
	Inhibitor4	Capsid	W80,M96,E98,W117,H120,P122,P123, I124,I129,R132	Inhibit capsid NTD interaction	B	4E91		2013	[26]
	BMMP	Capsid	?	Inhibit gag-gag interaction	B			2011	[27]
	Compound3,4,5	Capsid	?	Inhibit capsid NTD interaction	Cell-free			2013	[28]
	Compound27	Capsid	?	Inhibit capsid NTD interaction	Cell-free			2013	[29]
	CAP-1	Capsid	W23,V27,E28,A31,F32,V59,H62,A64, A65,I141	Inhibit capsid CTD interaction	B	2JPR		2007	[30]
	CAI-compound series	Capsid	V165,Y169,N183,L211,M215	Inhibit capsid CTD interaction	A,B,C,D,F,G, group O			2011	[31]
	Benzodiazepine series 33	Capsid	V27,A31,F32,V59,H62,A65,Y146	Inhibit capsid NTD interaction	Cell-free			2012	[32]
	Inhibitor3	Capsid	W23,V24.V27,E28,K30,A31,F32,S33, P34,V36,I37,F40,K56,V59,G60,G61,H62,A65,M66,K69,I134,K138,N139,I141,V142	Inhibit capsid NTD interaction	B	4INB		2013	[33]
	CAA	NC	V13,K14,F16,I24,A25,K26,R32,G35, W37,K38,Q45,M46,K47	Inhibit NC-RNA/DNA interaction	B	2M3Z		2013	[34]
	WDO-217	NC	?	Zinc ejection	B,HIV-2,SIV			2012	[35]

	Compound6,Compound8	NC	F16,R32,K34,W37,Q45,M46,K47	Inhibit NC(11-55)-RNA interaction	B			2012	[36]
	Compound45	NC	C49,T50,E51	Inhibit NC-oligonucleotide	B			1999	[37]
	SL3ligands	NC	zinc fingers(15-28,36-49)	Inhibit NC-RNA/DNA interaction	B			2012	[38]
	mONs	NC	zinc fingers	Inhibit NC-DNA interaction	Cell-free			2011	[39]
	SAMT	NC	zinc fingers	Inhibit NC-RNA/DNA	B			2010	[40]
	NV038	NC	zinc fingers	Zinc ejection	B,HIV-2,SIV			2010	[41]
	CO7	NC	zinc fingers	Inhibit NC-DNA interaction	Cell-free			2009	[42]
	Thioesters	NC	zinc fingers	Inhibit NC-RNA interaction	B			2004	[43]
	YS1332D	NC	zinc fingers	Inhibit NC-RNA interaction	B			2003	[44]
	DIBA	NC	zinc fingers	Zinc ejection	B,HIV-2,SIV			2001	[45]
	PATEs	NC	zinc fingers	Target zinc fingers	B			2001	[44]
	NSC 624151	NC	zinc fingers	Target zinc fingers	A,B,C,D,F,HIV-2,SIV			1996	[46]
	SRR-SB3	NC	zinc fingers	Target zinc fingers	B,HIV-2,SIV			1996	[47]
	NOBA	NC	zinc fingers	Target zinc fingers	Cell-free			1998	[48]
	Enantiomers	NC	zinc fingers	Inhibit NC-RNA interaction	Cell-free			2003	[49]
	2-Mercaptobenzamide Thioesters	NC	zinc fingers	Target zinc fingers	Cell-free			2005	[50]
	thiolcarbamates (TICAs),	NC	zinc fingers	Target zinc fingers	B			2002	[51]
	Azodicarbonamide (ADA)	NC	zinc fingers	Target zinc fingers	B,HIV-2		PhaseI/II (discontinued[52])	2000	[53]

Notations: (a) peptide inhibitors – amino acid sequences which were designed to inhibit HIV replication, (b) small organic molecule – low molecular weight organic compounds that were designed to bind gag proteins. Sequences of peptide inhibitors are:

(1) Matrix1[matrix sites: 47-59]: NPGLLETSEGCRQ,
(2) Capsid1[capsid sites:124-133]: IPVGEIYKRW,
(3) CAI: ITFEDLLDYYGPK(Bio[1])CL,
(4) CAC1M [capsid sites:175-193] SESAASSVKAWMTETLLVANTSS,
H8[capsid sites:158-176] KEPFRDYVDRFYKTLRAEQ,
(5) NYAD-201[capsid sites:178-192] AQEVKXWMTXTLLVA
(X= (S)-alpha- (2’-pentenyl)alanine),
(6) P-1[capsid sites: 181-192] VKNWMTETLLRQ,
(7) CP4: cyclo(D-Ala-Ile-Fpa-Arg-Tyr-Trp-D-Ala-D-Ala-Glu)-Lys
(8) HAGPIA [capsid sites:87-92] HAGPIA
(9) Gagp6[p6 sites:5-13]: PEPTAPPEE

Table S2. The summary of natural variants at drug binding sites of known gag inhibitors.

**Inhibitors**	**Ref**	**Target**	**HIV-1 subtypes**							
			**B(n=4131)**	**A1(n=1648)**	**C(n=2780)**	**D(n=443)**	**F1(n=35)**	**G(n=49)**	**01_AE(n=1714)**	**02_AG(n=62)**
CAI	[2]	Capsid		F169Y1.2,G183A1.2,G183N20.9,G183S2.4,E187D11.7	D187E14.1	N183A4.8,N183G7.0,N183T7.0,E187K2.7	G183N30.3,G183S3.0,D187E8.8,	G183N37.5,G183H2.1,G183S12.5,D187E10.4	Y169F1.3,N183H2.9	N183G4.9,N183H1.6,E187D14.8

CAC1/CAC1M,H8	[3]	Capsid	R154K29.1,K199R6.0,T216I2.4,T216S1.1,A194S12.3,T200S2.5,T210S2.7	K154R3.2,V191I17.1,K199R1.5,R203K8.1,A204G6.0,S200A3.7,S200G1.3,S200T10.1,T210S18.7	K154R26.3,V191I10.0,K199R1.2,R203K16.2,A204G12.9,T200N4.4,T200I5.0,T200S2.1,T200V2.4,T210S30.9,L211I1.4	R154K9.1,Q179T3.4,V191I4.3,I201L3.2,A204G4.1,T216S3.4,T200I4.1,T210S6.6	R154K33.3,V191I8.8,K199R2.9,K203R5.9,A204G5.9,L205M2.9,Q219R2.9,G220E2.9,A194S8.8,T200I2.9,A209G2.9,T210S18.2,L211I2.9	R154K46.8,Q179T2.1,V191I4.3,R203K31.9,A204G4.2,A204S2.1,T200N2.1,T200I2.1,T210S20.8	R154K12.7,V191I3.0,K199R1.2,K203R5.7,A204S2.7,S200A5.8,S200T3.4,T210S5.3	R154K27.9,V191I3.3,V191T1.6,K199R3.3,R203K33.3,A204G3.3,L205M3.3,T216S1.6,A194S3.3,S200A13.1,S200H1.6,S200I1.6,S200T14.8,T210S5.0
NYAD series	[5]	Capsid		F169Y1.2,G183A1.2,G183N20.9,G183S2.4	L211I1.4	N183A4.8,N183G7.0,N183T7.0	G183N30.3,G183S3.0,L211I2.9	G183N37.5,G183H2.1,G183S12.5	Y169F1.3,N183H2.9	N183G4.9,N183H1.6
P-1, P-2, P-3, P-4	[6]	Capsid	R154K29.1	K154R3.2,R203K8.1	K154R26.3,R203K16.2	R154K9.1,	R154K33.3,T188S5.9,K203R5.9	R154K46.8,T188S2.1,R203K31.9	R154K12.7,K203R5.7	R154K27.9,R203K33.3
Bevirimat analogs (C-28, C-30)	[15]	Capsid	Q6H1.9,V7A20.8,V7I1.7,V7M6.2,V7T1.1,T8S1.3	Q6H1.8,V7A16.5,V7I1.9,V7L1.4,Q8N1.3,Q8H3.1	Q6R1.0,Q6L1.7,A7T15.5,A7V22.7,N8Q1.1,N8G14.1,N8S7.1,N8T1.5	A1T2.3,A7V18.8,T8N15.4,T8S8.8	Q6K5.9,A7I3.0,A7L3.0,A7T6.1,A7V42.4,T8A3.3,T8N3.3,T8Q16.7,T8S6.7	Q6N2.1,Q6H10.4,Q6K2.1,A7I2.1,A7M2.1,A7V10.4,S8N2.1,S8T16.7	Q6N7.5,Q6H30.3,Q6K1.3,Q6S2.7,A7T4.0,A7V28.1	V7A31.1,V7T1.6,Q8H16.4
CP4	[7]	Capsid	T148A2.1,T148I2.5,T148S3.6,T148V22.3,A208G15.6,T216I2.4,T216S1.1	G208A1.6	V148T1.8,G208A5.5,L211I1.4	A208Q2.0,A208G38.9,T216S3.4	V148T2.9,G208A5.9,L211I2.9	V148T4.2,D152G2.1	V148I2.5,V148T10.2	V148T18.0,G208A1.6,T216S1.6
Compound 7	[11]	Matrix	K76R42.8,T81A9.1,T81L1.0,K98R1.0	R76K36.7,T81A4.5	R22K5.0,R76I3.8,R76K41.5,R76V1.3	K76R7.2,K98Q3.4	R22K8.8,W36R2.9,R76K38.2,T81A3.0,T81L6.1	L21M2.1,K76R39.6,T81A12.5,T81P2.1,K98Q6.4,K98T4.3	K76R17.4,T81A9.2,T81L4.1,K98Q1.6	K76R18.0,T81A3.3
TD1,TD2, TD3	[12]	Matrix	K26R8.2,K26N2.3,K26S2.1,L75I4.9,L75F7.5,K76R42.8	K26R6.6,K26N1.8,K26S1.1,L75I12.8,L75F4.5,L75V1.8,R76K36.7	R22K5.0,K26R1.7,E73K5.1,L75I2.8,L75F8.5,R76I3.8,R76K41.5,R76V1.3	N26R6.7,N26G3.0,N26H12.0,N26K24.2,N26S24.2,K27Q2.0,E73K4.8,I75L24.9,I75M2.0,I75V2.0,K76R7.2	R22K8.8,K26R2.9,W36R2.9,L75I5.9,L75F2.9,R76K38.2	L21M2.1,K26R39.6,K27N4.2,E73Q4.2,L75I42.6,K76R39.6	K26R7.7,L75I6.1,L75F1.6,L75Y1.2,L75V4.2,K76R17.4	K26R1.6,K26N3.3,K26S3.3,L75I4.9,L75V1.6,K76R18.0
PF-3450074	[24]	Capsid	T107S2.8			T107S2.9	T107A4.2,T107S6.2	T107S1.2		

I-XW-053	[23]	Capsid	K170R4.5,T171V2.6,S178T10.4,E180D36.1	S33N4.7,F169Y1.2,T171A4.7,T171C9.8,T171I1.2,T171V7.7,T178S17.4,E180D25.5,E180P1.1,G183A1.2,G183N20.9,G183S2.4	S33N15.9,V36I11.4,K170R3.2,T171A1.2,T171V4.0,T178S15.1,D180E38.4	S33N7.7,T171V8.8,S178T5.0,Q179T3.4,D180N2.7,D180E11.1,N183A4.8,N183G7.0,N183T7.0	S33N2.9,V36I2.9,K170R9.1,T171A9.1,T171C3.0,T171V6.1,T178S26.5,E180D11.8,G183N30.3,G183S3.0	S33N6.2,T171A4.2,T171C2.1,T171V2.1,T178S10.4,Q179T2.1,E180D29.2,G183N37.5,G183H2.1,G183S12.5	N33S2.7,Y169F1.3,T178S16.1,E180D1.6,N183H2.9	E35K1.6,T171A1.6,T171V1.6,T178S3.3,E180D13.1,N183G4.9,N183H1.6
BM4	[25]	Capsid	V27I25.1,	I27V2.3,K30R13.3,S33N4.7,I58V6.4	I27V13.9,S33N15.9	I27V17.0,S33N7.7	W23M2.9,I27V33.3,K30R2.9,S33N2.9	V27I16.7,K30N2.1,S33N6.2	V27I7.0,N33S2.7	I27V3.3,K30R1.6,I58V1.6
BD3	[25]	Capsid	V27I25.1,	I27V2.3,I58V6.4,V59I1.9	I27V13.9,V36I11.4,V59I3.0	I27V17.0	W23M2.9,I27V33.3,V36I2.9	V27I16.7	V27I7.0,G61D2.0	I27V3.3,I58V1.6
Inhibitor4	[26]	Capsid	M96I8.6,M96L3.4,M96V3.5,E98D11.0,N120H17.9,N120S23.2,P123A1.8,I124V3.6,R132K5.8	M96I8.9,M96L12.2,S120N7.5,S120G16.0,I124F1.8,I124V2.4,R132G2.0,R132K5.5	M96I20.2,M96L5.1,M96S1.1,E98D6.0,S120A3.1,S120N16.6,S120G19.4,S120H1.2,P123A2.2,I124V35.7	M96I15.2,E98D7.0,S120A2.5,S120N38.0,S120G1.1,S120H2.3,I124V6.3,R132K7.3	M96I32.4,M96L2.9,E98D2.9,S120A2.9,S120G14.7,P122Q2.9,V124I2.9,I129M2.9,R132G2.9,R132K2.9	I96M2.1,E98D20.8,E98G2.1,W117R2.1,S120N10.6,S120G6.4,P123A2.1,I124V10.4	M96I13.5,M96L8.5,E98D2.1,N120G10.8,N120S35.8,P123A8.4,P123S4.1,I124V1.8	M96I1.6,M96L1.6,E98D1.6,S120N1.6,S120G4.9,I124T1.6,I124V14.8,I129M1.6,
CAP-1	[30]	Capsid	V27I25.1,	I27V2.3,A31G24.5,V59I1.9,I141L5.6	I27V13.9,A31N5.6,A31G16.2,V59I3.0	I27V17.0,E28K2.9,A31G6.3	W23M2.9,I27V33.3,E28G2.9,A31G2.9	V27I16.7,A31G2.1,A31S2.1	V27I7.0,G31S1.1,A64G1.5	I27V3.3,A31N1.6,A31G11.5
CAI-compound series	[31]	Capsid		F169Y1.2,G183A1.2,G183N20.9,G183S2.4	L211I1.4	N183A4.8,N183G7.0,N183T7.0	G183N30.3,G183S3.0,L211I2.9	G183N37.5,G183H2.1,G183S12.5	Y169F1.3,N183H2.9	N183G4.9,N183H1.6
Benzodiazepine series 33	[32]	Capsid	V27I25.1	I27V2.3,A31G24.5,V59I1.9,S146N1.9	I27V13.9,A31N5.6,A31G16.2,V59I3.0	I27V17.0,A31G6.3	I27V33.3,A31G2.9	V27I16.7,A31G2.1,A31S2.1,S146C4.2	V27I7.0,G31S1.1,S146R1.9,S146K1.6	I27V3.3,A31N1.6,A31G11.5
Inhibitor3	[33]	Capsid		K30R13.3,A31G24.5,S33N4.7,V59I1.9,N139H5.6,I141L5.6	A31N5.6,A31G16.2,S33N15.9,V36I11.4,V59I3.0	E28K2.9,A31G6.3,S33N7.7,I134V1.4	W23M2.9,E28G2.9,K30R2.9,A31G2.9,S33N2.9,V36I2.9	K30N2.1,A31G2.1,A31S2.1,S33N6.2,N139H2.1	G31S1.1,N33S2.7,G61D2.0,N139H1.2	K30R1.6,A31N1.6,A31G11.5
CAA	[34]	NC	V13I15.3,I24L11.6,I24T1.5,I24V1.8,K26R48.5	R26K6.0	V13I33.7,F16Y1.7,I24L22.7,R26K25.0	I13L13.2,I13V9.1,I24L14.5,I24T13.3,K26R31.7	V13I35.3,I24L11.8,I24V2.9,K26R42.4	K14R2.1,F16Y2.1,L24I2.1,R26K2.1,Q45L2.1,M46I2.1,K47R18.8	R26K3.6	I13L1.6,I13V1.6,L24V1.6,R26K1.6,Q45L1.6
Compound 6,Compound8	[36]	NC	K34R17.9	F16Y1.7,K34R11.9	R34K40.1	K34R2.9	F16Y2.1,K34R6.2,Q45L2.1,M46I2.1,K47R18.8	K34R2.9	R34K29.3,Q45L1.6	

Compound 45	[37]	NC	T50N4.7,T50I2.2,T50S4.0,E51G1.6	T50D1.3,T50E1.4,E51D1.2	T50N3.1		T50N2.9,T50E2.9,T50P2.9	T50M2.1,T50S2.1,E51D2.1,E51Q2.1	T50N4.6	T50N3.3,E51V1.6
Bevirimat	[14]	CA-p2	Q6H1.9,V7A20.8,V7I1.7,V7M6.2,V7T1.1,N9Q1.6,N9G8.4,N9S6.7,N9T2.6,I13M2.3,I13V14.1	Q6H1.8,V7A16.5,V7I1.9,V7L1.4,H9N11.5,H9Q29.5,H9G1.1,I13V7.8	Q6R1.0,Q6L1.7,A7T15.5,A7V22.7,N9Q4.9,N9H6.0,N9S20.7,M13L12.2	A7V18.8,N9Q3.4,N9G13.6,N9K12.7,N9S14.7,N9T2.3,N9V4.8,I13A1.6,I13L6.3,I13M3.6,I13V18.8	Q6K5.9,A7I3.0,A7L3.0,A7T6.1,A7V42.4,N9G3.0,N9H12.1,N9K6.1,N9S21.2,V13I44.1,V13L2.9	Q6N2.1,Q6H10.4,Q6K2.1,A7I2.1,A7M2.1,A7V10.4,G9N4.3,G9D2.1,G9S2.1,G9T2.1,I13A2.2,I13M2.2,I13V17.8	Q6N7.5,Q6H30.3,Q6K1.3,Q6S2.7,A7T4.0,A7V28.1,H9N5.6,H9Q35.9,H9G2.6,H9P1.3,H9S1.5,I13M1.3,I13V15.4	V7A31.1,V7T1.6,Q9N3.3,I13V34.4

Inhibitor names, references and targets are indicated in the first three columns. The proportions of natural variations for subtype B, A1, C, D, F1, G, 01AE and 02AG were summarized from the 4th to 11th columns. Natural polymorphisms for each inhibitor are summarized for each subtype and they are annotated in the form of "wildtype+position+mutation+proportion". For instance, "R154K29.1" indicates the most prevalent amino acid R at position 154 switches to amino acid K, with the proportion 29.1% in sequence dataset. Note that the most prevalent amino acid is defined as wildtype in our analysis.

References

1. Niedrig M, Gelderblom HR, Pauli G, Marz J, Bickhard H, Wolf H, Modrow S: **Inhibition of infectious human immunodeficiency virus type 1 particle formation by Gag protein-derived peptides.** *J Gen Virol* 1994, **75 (Pt 6):**1469-1474.
2. Ternois F, Sticht J, Duquerroy S, Krausslich HG, Rey FA: **The HIV-1 capsid protein C-terminal domain in complex with a virus assembly inhibitor.** *Nat Struct Mol Biol* 2005, **12:**678-682.
3. Bocanegra R, Nevot M, Domenech R, Lopez I, Abian O, Rodriguez-Huete A, Cavasotto CN, Velazquez-Campoy A, Gomez J, Martinez MA, et al: **Rationally designed interfacial peptides are efficient in vitro inhibitors of HIV-1 capsid assembly with antiviral activity.** *PLoS One* 2011, **6:**e23877.
4. Bhattacharya S, Zhang H, Debnath AK, Cowburn D: **Solution structure of a hydrocarbon stapled peptide inhibitor in complex with monomeric C-terminal domain of HIV-1 capsid.** *J Biol Chem* 2008, **283:**16274-16278.
5. Zhang H, Curreli F, Zhang X, Bhattacharya S, Waheed AA, Cooper A, Cowburn D, Freed EO, Debnath AK: **Antiviral activity of alpha-helical stapled peptides designed from the HIV-1 capsid dimerization domain.** *Retrovirology* 2011, **8:**28.
6. Domenech R, Bocanegra R, Gonzalez-Muniz R, Gomez J, Mateu MG, Neira JL: **Larger helical populations in peptides derived from the dimerization helix of the capsid protein of HIV-1 results in peptide binding toward regions other than the "hotspot" interface.** *Biomacromolecules* 2011, **12:**3252-3264.

7. Dewan V, Liu T, Chen KM, Qian Z, Xiao Y, Kleiman L, Mahasenan KV, Li C, Matsuo H, Pei D, Musier-Forsyth K: **Cyclic peptide inhibitors of HIV-1 capsid-human lysyl-tRNA synthetase interaction.** *ACS Chem Biol* 2012, **7:**761-769.
8. Vajdos FF, Yoo S, Houseweart M, Sundquist WI, Hill CP: **Crystal structure of cyclophilin A complexed with a binding site peptide from the HIV-1 capsid protein.** *Protein Sci* 1997, **6:**2297-2307.
9. Im YJ, Kuo L, Ren X, Burgos PV, Zhao XZ, Liu F, Burke TR, Jr., Bonifacino JS, Freed EO, Hurley JH: **Crystallographic and functional analysis of the ESCRT-I /HIV-1 Gag PTAP interaction.** *Structure* 2010, **18:**1536-1547.
10. Zentner I, Sierra LJ, Maciunas L, Vinnik A, Fedichev P, Mankowski MK, Ptak RG, Martin-Garcia J, Cocklin S: **Discovery of a small-molecule antiviral targeting the HIV-1 matrix protein.** *Bioorg Med Chem Lett* 2013, **23:**1132-1135.
11. Zentner I, Sierra LJ, Fraser AK, Maciunas L, Mankowski MK, Vinnik A, Fedichev P, Ptak RG, Martin-Garcia J, Cocklin S: **Identification of a small-molecule inhibitor of HIV-1 assembly that targets the phosphatidylinositol (4,5)-bisphosphate binding site of the HIV-1 matrix protein.** *ChemMedChem* 2013, **8:**426-432.
12. Alfadhli A, McNett H, Eccles J, Tsagli S, Noviello C, Sloan R, Lopez CS, Peyton DH, Barklis E: **Analysis of small molecule ligands targeting the HIV-1 matrix protein-RNA binding site.** *J Biol Chem* 2013, **288:**666-676.
13. Smith PF, Ogundele A, Forrest A, Wilton J, Salzwedel K, Doto J, Allaway GP, Martin DE: **Phase I and II study of the safety, virologic effect, and pharmacokinetics/pharmacodynamics of single-dose 3-o-(3',3'-dimethylsuccinyl)betulinic acid (bevirimat) against human immunodeficiency virus infection.** *Antimicrob Agents Chemother* 2007, **51:**3574-3581.
14. Li F, Goila-Gaur R, Salzwedel K, Kilgore NR, Reddick M, Matallana C, Castillo A, Zoumplis D, Martin DE, Orenstein JM, et al: **PA-457: a potent HIV inhibitor that disrupts core condensation by targeting a late step in Gag processing.** *Proc Natl Acad Sci U S A* 2003, **100:**13555-13560.
15. Nguyen AT, Feasley CL, Jackson KW, Nitz TJ, Salzwedel K, Air GM, Sakalian M: **The prototype HIV-1 maturation inhibitor, bevirimat, binds to the CA-SP1 cleavage site in immature Gag particles.** *Retrovirology* 2011, **8:**101.
16. Vijay Baichwal HA, Brita Brown, Rena McKinnon, Kraig Yager,Vijay Kumar, David Gerrish, Mark Anderson and Robert Carlson: **Anti-viral Characterization in vitro of a Novel Maturation Inhibitor, MPC-9055.** In *Program & Abstracts of the 16th Conference on Retroviruses and Opportunistic Infections; Montreal, Canada.* . 2009. Abstract 561
17. Singh IP, Bodiwala HS: **Recent advances in anti-HIV natural products.** *Nat Prod Rep* 2010, **27:**1781-1800.
18. Kilgore N. RM, Zuiderhof M., Stanley D., Nitz T., Bullock P., Allaway G., Martin D: **Characterization of PA1050040, a second generation HIV-1 maturation inhibitor.** In *IAS 2007, 4th IAS Conference On HIV Pathogenesis, Treatment and Prevention*. Sydney, Australia; 2007. Abstract MOPDX05.

19. Vijay Kumar DG, Christophe Hoarau, Kraig M. Yager, Harry Austin, Rena McKinnon, Brita Brown, Irene Dorweiler, Vijay Baichwal, Damon Papac, Chad Bradford, Scott Patton, Katrina Bulka, Lynn DeMie and Robert Carlson: **Next Generation Orally Bioavailable HIV-1 Maturation Inhibitors.** In *239th ACS National Meeting & Exposition; San Francisco,CA.* 2010
20. Blair WS, Cao J, Fok-Seang J, Griffin P, Isaacson J, Jackson RL, Murray E, Patick AK, Peng Q, Perros M, et al: **New small-molecule inhibitor class targeting human immunodeficiency virus type 1 virion maturation.** *Antimicrob Agents Chemother* 2009, **53:**5080-5087.
21. Waki K, Durell SR, Soheilian F, Nagashima K, Butler SL, Freed EO: **Structural and functional insights into the HIV-1 maturation inhibitor binding pocket.** *PLoS Pathog* 2012, **8:**e1002997.
22. Coric P, Turcaud S, Souquet F, Briant L, Gay B, Royer J, Chazal N, Bouaziz S: **Synthesis and biological evaluation of a new derivative of bevirimat that targets the Gag CA-SP1 cleavage site.** *Eur J Med Chem* 2013, **62:**453-465.
23. Kortagere S, Madani N, Mankowski MK, Schon A, Zentner I, Swaminathan G, Princiotto A, Anthony K, Oza A, Sierra LJ, et al: **Inhibiting early-stage events in HIV-1 replication by small-molecule targeting of the HIV-1 capsid.** *J Virol* 2012, **86:**8472-8481.
24. Blair WS, Pickford C, Irving SL, Brown DG, Anderson M, Bazin R, Cao J, Ciaramella G, Isaacson J, Jackson L, et al: **HIV capsid is a tractable target for small molecule therapeutic intervention.** *PLoS Pathog* 2010, **6:**e1001220.
25. Lemke CT, Titolo S, von Schwedler U, Goudreau N, Mercier JF, Wardrop E, Faucher AM, Coulombe R, Banik SS, Fader L, et al: **Distinct effects of two HIV-1 capsid assembly inhibitor families that bind the same site within the N-terminal domain of the viral CA protein.** *J Virol* 2012, **86:**6643-6655.
26. Goudreau N, Lemke CT, Faucher AM, Grand-Maitre C, Goulet S, Lacoste JE, Rancourt J, Malenfant E, Mercier JF, Titolo S, Mason SW: **Novel Inhibitor Binding Site Discovery on HIV-1 Capsid N-Terminal Domain by NMR and X-ray Crystallography.** *ACS Chem Biol* 2013, **8:**1074-1082.
27. Urano E, Kuramochi N, Ichikawa R, Murayama SY, Miyauchi K, Tomoda H, Takebe Y, Nermut M, Komano J, Morikawa Y: **Novel postentry inhibitor of human immunodeficiency virus type 1 replication screened by yeast membrane-associated two-hybrid system.** *Antimicrob Agents Chemother* 2011, **55:**4251-4260.
28. Fader LD, Landry S, Morin S, Kawai SH, Bousquet Y, Hucke O, Goudreau N, Lemke CT, Bonneau P, Titolo S, et al: **Optimization of a 1,5-dihydrobenzo[b][1,4]diazepine-2,4-dione series of HIV capsid assembly inhibitors 1: Addressing configurational instability through scaffold modification.** *Bioorg Med Chem Lett* 2013, **23:**3396-3400.
29. Fader LD, Landry S, Goulet S, Morin S, Kawai SH, Bousquet Y, Dion I, Hucke O, Goudreau N, Lemke CT, et al: **Optimization of a 1,5-dihydrobenzo[b][1,4]diazepine-2,4-dione series of HIV capsid assembly inhibitors 2: Structure-activity relationships (SAR) of the C3-phenyl moiety.** *Bioorg Med Chem Lett* 2013, **23:**3401-3405.

30. Kelly BN, Kyere S, Kinde I, Tang C, Howard BR, Robinson H, Sundquist WI, Summers MF, Hill CP: **Structure of the antiviral assembly inhibitor CAP-1 complex with the HIV-1 CA protein.** *J Mol Biol* 2007, **373:**355-366.
31. Curreli F, Zhang H, Zhang X, Pyatkin I, Victor Z, Altieri A, Debnath AK: **Virtual screening based identification of novel small-molecule inhibitors targeted to the HIV-1 capsid.** *Bioorg Med Chem* 2011, **19:**77-90.
32. Tremblay M, Bonneau P, Bousquet Y, DeRoy P, Duan J, Duplessis M, Gagnon A, Garneau M, Goudreau N, Guse I, et al: **Inhibition of HIV-1 capsid assembly: optimization of the antiviral potency by site selective modifications at N1, C2 and C16 of a 5-(5-furan-2-yl-pyrazol-1-yl)-1H-benzimidazole scaffold.** *Bioorg Med Chem Lett* 2012, **22:**7512-7517.
33. Goudreau N, Coulombe R, Faucher AM, Grand-Maitre C, Lacoste JE, Lemke CT, Malenfant E, Bousquet Y, Fader L, Simoneau B, et al: **Monitoring binding of HIV-1 capsid assembly inhibitors using (19)F ligand-and (15)N protein-based NMR and X-ray crystallography: early hit validation of a benzodiazepine series.** *ChemMedChem* 2013, **8:**405-414.
34. Goudreau N, Hucke O, Faucher AM, Grand-Maitre C, Lepage O, Bonneau PR, Mason SW, Titolo S: **Discovery and Structural Characterization of a New Inhibitor Series of HIV-1 Nucleocapsid Function: NMR Solution Structure Determination of a Ternary Complex Involving a 2:1 Inhibitor/NC Stoichiometry.** *J Mol Biol* 2013.
35. Vercruysse T, Basta B, Dehaen W, Humbert N, Balzarini J, Debaene F, Sanglier-Cianferani S, Pannecouque C, Mely Y, Daelemans D: **A phenyl-thiadiazolylidene-amine derivative ejects zinc from retroviral nucleocapsid zinc fingers and inactivates HIV virions.** *Retrovirology* 2012, **9:**95.
36. Mori M, Schult-Dietrich P, Szafarowicz B, Humbert N, Debaene F, Sanglier-Cianferani S, Dietrich U, Mely Y, Botta M: **Use of virtual screening for discovering antiretroviral compounds interacting with the HIV-1 nucleocapsid protein.** *Virus Res* 2012, **169:**377-387.
37. Turpin JA, Song Y, Inman JK, Huang M, Wallqvist A, Maynard A, Covell DG, Rice WG, Appella E: **Synthesis and biological properties of novel pyridinioalkanoyl thiolesters (PATE) as anti-HIV-1 agents that target the viral nucleocapsid protein zinc fingers.** *J Med Chem* 1999, **42:**67-86.
38. Breuer S, Chang MW, Yuan J, Torbett BE: **Identification of HIV-1 inhibitors targeting the nucleocapsid protein.** *J Med Chem* 2012, **55:**4968-4977.
39. Avilov SV, Boudier C, Gottikh M, Darlix JL, Mely Y: **Characterization of the inhibition mechanism of HIV-1 nucleocapsid protein chaperone activities by methylated oligoribonucleotides.** *Antimicrob Agents Chemother* 2012, **56:**1010-1018.
40. Miller Jenkins LM, Ott DE, Hayashi R, Coren LV, Wang D, Xu Q, Schito ML, Inman JK, Appella DH, Appella E: **Small-molecule inactivation of HIV-1 NCp7 by repetitive intracellular acyl transfer.** *Nat Chem Biol* 2010, **6:**887-889.
41. Pannecouque C, Szafarowicz B, Volkova N, Bakulev V, Dehaen W, Mely Y, Daelemans D: **Inhibition of HIV-1 replication by a bis-thiadiazolbenzene-1,2-diamine that chelates zinc ions from retroviral nucleocapsid zinc fingers.** *Antimicrob Agents Chemother* 2010, **54:**1461-1468.

42. Shvadchak V, Sanglier S, Rocle S, Villa P, Haiech J, Hibert M, Van Dorsselaer A, Mely Y, de Rocquigny H: **Identification by high throughput screening of small compounds inhibiting the nucleic acid destabilization activity of the HIV-1 nucleocapsid protein.** *Biochimie* 2009, **91:**916-923.
43. Srivastava P, Schito M, Fattah RJ, Hara T, Hartman T, Buckheit RW, Jr., Turpin JA, Inman JK, Appella E: **Optimization of unique, uncharged thioesters as inhibitors of HIV replication.** *Bioorg Med Chem* 2004, **12:**6437-6450.
44. Schito ML, Goel A, Song Y, Inman JK, Fattah RJ, Rice WG, Turpin JA, Sher A, Appella E: **In vivo antiviral activity of novel human immunodeficiency virus type 1 nucleocapsid p7 zinc finger inhibitors in a transgenic murine model.** *AIDS Res Hum Retroviruses* 2003, **19:**91-101.
45. Sharmeen L, McQuade T, Heldsinger A, Gogliotti R, Domagala J, Gracheck S: **Inhibition of the early phase of HIV replication by an isothiazolone, PD 161374.** *Antiviral Res* 2001, **49:**101-114.
46. Rice WG, Baker DC, Schaeffer CA, Graham L, Bu M, Terpening S, Clanton D, Schultz R, Bader JP, Buckheit RW, Jr., et al: **Inhibition of multiple phases of human immunodeficiency virus type 1 replication by a dithiane compound that attacks the conserved zinc fingers of retroviral nucleocapsid proteins.** *Antimicrob Agents Chemother* 1997, **41:**419-426.
47. Witvrouw M, Balzarini J, Pannecouque C, Jhaumeer-Laulloo S, Este JA, Schols D, Cherepanov P, Schmit JC, Debyser Z, Vandamme AM, et al: **SRR-SB3, a disulfide-containing macrolide that inhibits a late stage of the replicative cycle of human immunodeficiency virus.** *Antimicrob Agents Chemother* 1997, **41:**262-268.
48. Huang M, Maynard A, Turpin JA, Graham L, Janini GM, Covell DG, Rice WG: **Anti-HIV agents that selectively target retroviral nucleocapsid protein zinc fingers without affecting cellular zinc finger proteins.** *J Med Chem* 1998, **41:**1371-1381.
49. Mayasundari A, Rice WG, Diminnie JB, Baker DC: **Synthesis, resolution, and determination of the absolute configuration of the enantiomers of cis-4,5-dihydroxy-1,2-dithiane 1,1-dioxide, an HIV-1NCp7 inhibitor.** *Bioorg Med Chem* 2003, **11:**3215-3219.
50. Jenkins LM, Byrd JC, Hara T, Srivastava P, Mazur SJ, Stahl SJ, Inman JK, Appella E, Omichinski JG, Legault P: **Studies on the mechanism of inactivation of the HIV-1 nucleocapsid protein NCp7 with 2-mercaptobenzamide thioesters.** *J Med Chem* 2005, **48:**2847-2858.
51. Goel A, Mazur SJ, Fattah RJ, Hartman TL, Turpin JA, Huang M, Rice WG, Appella E, Inman JK: **Benzamide-based thiolcarbamates: a new class of HIV-1 NCp7 inhibitors.** *Bioorg Med Chem Lett* 2002, **12:**767-770.
52. Dau B, Holodniy M: **Novel targets for antiretroviral therapy: clinical progress to date.** *Drugs* 2009, **69:**31-50.
53. Rice WG, Turpin JA, Huang M, Clanton D, Buckheit RW, Jr., Covell DG, Wallqvist A, McDonnell NB, DeGuzman RN, Summers MF, et al: **Azodicarbonamide inhibits HIV-1 replication by targeting the nucleocapsid protein.** *Nat Med* 1997, **3:**341-345.
