# Supplementary material for: Functional conservation of HIV-1 Gag: implications for rational drug design
Source: Retrovirology. 2013 Oct 31;10:126. doi: 10.1186/1742-4690-10-126 (PMC4228425; doi:10.1186/1742-4690-10-126)
Supplement: Additional file 2 — Figure S1. The distribution of natural variations of full-length gag in 8 HIV-1 subtypes. Figure S2. The surface representation of full-length gag in 8 HIV-1 subtypes and CRFs. Figure S3. The surface representation of five drug binding pockets in 8 HIV-1 subtypes. Figure S3. The surface representation of full-length gag in 8 HIV-1 subtypes and CRFs. Figure S4. The structure of capsid hexamer superimposed with 8 crystalized inhibitors. Figure S5. The surface representation of conserved regions in HIV-1 capsid Figure S6. The surface representation of conserved regions in HIV-1 nucleocapsid. [file 1742-4690-10-126-S2.pdf]

Additional file 2: Figures

Title:

Functional conservation of HIV-1 gag: implications for rational drug design

Authors and Affiliations

Guangdi Li[1], Jens Verheyen[2], Soo-Yon Rhee[1,3], Arnout Voet[4], Anne-Mieke Vandamme[1,5], Kristof Theys[1*]

*Corresponding author: Kristof Theys Kristof.Theys@rega.kuleuven.be

1 Rega Institute, Department of Microbiology and Immunology, KU Leuven, Leuven, Belgium
2 Institute of Virology, University hospital, University Duisburg-Essen, Essen, Germany
3 Division of Infectious Diseases, Department of Medicine, Stanford University, Stanford, California, USA
4 Zhang IRU, RIKEN Institute Laboratories, Hirosawa 2-1, Wako-shi, Saitama, Japan
5 Centro de Malária e Outras Doenças Tropicais and Unidade de Microbiologia, Instituto de Higiene e Medicina Tropical, Universidade Nova de Lisboa, Lisboa, Portugal

Email: Guangdi Li liguangdi.research@gmail.com
Jens Verheyen jens.verheyen@uk-essen.de
Soo-Yon Rhee syrhee@stanford.edu
Arnout Voet arnout.voet@fys.kuleuven.be
Anne-Mieke Vandamme annemie.vandamme@uz.kuleuven.ac.be
Kristof Theys Kristof.Theys@rega.kuleuven.be

Figure S1. The distribution of natural polymorphisms at 500 gag positions on 8 HIV-1 subtypes. HXB2 indices at each individual protein are shown in the first column, followed by HXB2 indices in the full-length gag. Drug binding positions are marked with red stars. The colors in the second column distinguish the gag domains: light green for matrix (position: 1-132), light blue for capsid (133-363), dark green for p2 (364-377) and p1 (433-448), dark blue for nucleocapsid (378-432) and gray for p6 (449-500). The remaining columns list the consensus amino acid for each subtype followed its natural variation(s) and the corresponding proportion(s) in blue (prevalence above 5%) and orange (prevalence at or below 5%).

Figure S2: Surface representation of conservation index of full-length gag in 8 HIV-1 subtypes. 4 gag proteins (monomers) and 2 spacer peptides are annotated and displayed in schematic view at the top. Drug binding sites (red) are mapped onto HIV-1 gag protein structures. For each subtype figure, surface spectrum colors indicate each position's CI, from the most conserved (blue CI = 0) to the least conserved positions (pink CI $\geq$ 0.1). Crystallized inhibitors are shown in sticks view inside their binding pockets. Visualization software: PyMOL V1.5 (http://www.pymol.org/).

Figure S3: Surface representation of five drug binding pockets in 8 HIV-1 subtypes. For each subtype figure, surface spectrum colors indicate each position's CI, from the most conserved (blue CI = 0) to the least conserved positions (pink CI ≥ 0.1). Crystallized inhibitors are shown in sticks view inside their binding pockets. Visualization software: PyMOL V1.5 (http://www.pymol.org/).

Figure S4: The structure of capsid hexamer superimposed with 8 crystallized inhibitors. The capsid hexamer structure is shown in the top, side and bottom views from Fig S1(A) to S1(C). The drug binding pockets are annotated in each figure. Three figures on the left side show the N-terminal domains (NTD, position: 1-146) and C-terminal domains (CTD, position: 151-231), colored blue and pink, respectively. Red circles indicate drug binding pockets, whose targets are within the interfaces of NTD-NTD, NTD-CTD or CTD-CTD interactions. Three figures on right side map the CIs to the structure and visualize the conservation of the drug binding pockets. It shows that drug binding pocket 1 in NTD is situated on the NTD-CTD interaction interface, drug binding pocket 2 in NTD is situated on the NTD-NTD interaction interface, drug binding pocket 3 in CTD is situated on the NTD-CTD interaction interface, and drug binding pocket 4 is inside NTD. Note that drug binding pockets 1 and 3 are situated on the opposite sides of the same NTD-CTD interface. The superimposed crystallized inhibitors were mapped onto the capsid hexamer using PyMOL V1.5 (PDB: 3H4E). Visualization software: PyMOL V1.5 (http://www.pymol.org/). Inhibitor references are available in Additional file 1.

Figure S5: Conserved NTD and CTD domains in HIV-1 capsid. The capsid hexamer structure is shown in top (A), side (B) and bottom (C) views. Conserved NTD-NTD interaction domains are colored yellow (capsid positions: 30-70, gag positions: 162-202). Conserved NTD-CTD interaction domains are colored red (capsid positions: 155-176, gag positions: 287-308). PDB: 3H4E. Visualization software: PyMOL V1.5 (http://www.pymol.org/).

Figure S6: Conserved zinc-finger domains in HIV-1 nucleocapsid. The structures of nucleocapsid–RNA and nucleocapsid–inhibitor complexes are shown in top (A), side (B) and bottom (C) views. The first zinc-finger domain is colored red (nucleocapsid positions: 14-29, gag positions: 389-404) and the second zinc-finger domain (nucleocapsid positions: 35-50, gag positions: 410-425) are colored orange. PDB: 1A1T, 2M3Z. Visualization software: PyMOL V1.5 (http://www.pymol.org/).

Figure S5 and S6 visualizes the conserved regions identified as three minimum conserved regions using our conservation analysis (Figure 2).

Figure S1. Natural variations of gag positions in 8 major HIV-1 subtypes and CRFs

gag	B	A1	C	D	F1	G	01_AE	02_AG
1	M	M I.9	M	M	M	M	M	M
2	G	G	G	G	G	G	G	G
3	A	A	A	A	A	A	A	A
4	R	R	R S2.1	R S5.7	R	R	R	R
5	A	A	A	A	A	A	A	A
6	S	S	S	S	S	S	S	S
7	V I16.2	V I6.6	I V27.5	V	V	V	V I13.0	V I16.4
8	L I4.0	L	L	L I3.8	L	L	L	L
9	S R4.6	S T1.5. R1.3	R K11.6	S R5.2	S	S	S T13.9R1.7. K1.1	S T14.8
10	G A2.0	G	G	G	G	G	G	G
11	G E2.6. A1.2	G	G E42.6K1.4	G E5.7	G	G	G R1.9. E1.9	G
12	E K25.4Q13.5D2.5. G1.5	K	K N3.5	K R11.3S4.8. Q4.1. E3.4	K	K	K Q.9	K R8.2
13	L	L	L	L	L	L	L	L
14	D	D E4.5. N.9	D	D	D	D	D	D
15	R K25.7Q3.3. S1.3. A.9	A T7.4. S4.1. K4.1. M1.8. Q1.3. E1.3	K T27.0A15.8R5.6. N3.9	E D9.5. A6.3.	A D17.6	K A39.6	A T3.7. S2.3. R1.2	A S23.0
16	W	W	W	W	W	W	W	W
17	E	E	E	E	E	E	E	E
18	K R7.7. G2.1	K R2.0	K R28.1	K R19.9	K	K	K R9.3	K R9.8
19	I	I T1.3	I	I	I	I	I	I
20	R	R Q3.0. E1.6	R K18.1S1.5	R Q24.0	R	R	R	R
* 21	L	L	L	L	L	L	L	L
* 22	R	R	R K5.0	R	R	R	R	R
23	P	P	P	P	P	P	P	P
24	G	G	G	G	G	G	G	G
25	G	G	G S2.3	G E4.8	G	G	G	G
* 26	K R8.1. N2.3. S2.1	K R6.6. N1.8. S1.1	K R1.7	N K23.8S23.8H11.8R6.6. G2.9	K	K R39.6	K R7.6	K
* 27	K	K	K	K Q2.0.	K	K	K	K
28	K Q21.7R10.8T2.2	K Q18.5R3.4. T1.7	H K16.4Q11.9C11.3R10.1S2.6. T1.7	K R16.3Q6.8	K Q26.5	K Q39.6R10.4	K R3.7. Q1.2	K Q18.0
29	Y	Y	Y	Y	Y	Y	Y	Y
30	K R32.7Q9.8	R K8.6. Q1.2	M R13.7Q6.5. K5.6	K R36.7	R K20.6	K R45.8	R K33.6Q5.8	R K13.1
31	L	M L45.9 I3.8	L I15.1M9.5	L	M L23.5	M L33.3	M L30.4 I9.5	L
32	K	K	K	K R5.9	K	K	K	K
* 33	H	H	H	H	H	H	H	H
34	I L22.8V2.5	L I3.7	L I23.7	I L31.7	L	L I16.7	L I14.5	L I16.4
35	V I1.5	V	V I4.9	V	V I41.2	V	V I8.1	V
* 36	W	W	W	W	W	W	W	W
37	A	A	A	A	A	A	A	A
38	S	S G2.1	S	S G2.0	S	S	S	S
39	R	R	R K1.1	R K2.7	R	R	R	R
40	E	E	E	E Q4.5	E	E	E	E
41	L	L M1.9	L	L	L	L M10.4	L V1.3	L V13.1
42	E	E D8.1	E D2.4. G1.9	E	E	E D14.6	E D4.1	E D14.8
43	R	R K2.5	R K14.1	R	R	R	R	R
44	F Y1.5	F	F	F Y3.4	F	F	F	F
45	A	A	A S1.2	A	A	A	A	A
46	V L9.0. I3.2	L V2.4. I.9	L	L I24.2V1.4	L I23.5V20.6	L	L I14.1V8.1	L I8.2
47	N	N	N D4.7	N D4.8	D	N	N D3.4	N
48	P	P S2.1	P S2.9	P	P	P S20.8	P	P
49	G S6.0	S G19.7N2.5	G D10.9S4.8	G S12.0	G	G D33.3	G E2.1	G S8.2
50	L	L	L	L P1.6	L	L	L F.10	L
51	L	L	L	L	L	L I10.4	L	L
52	E	E D1.9	E	E	E	E	E	E
53	T S2.3	T S.10	T S1.3	T S6.6	T	T	T S9.8	T S16.4
54	S A18.5T1.7. P1.3	A T44.2P3.7. K3.5. S2.0. R1.3	S A14.6E1.0. P.10	S T8.6. A7.2. P5.4. E4.1	S P26.5	A T16.7	A S6.3. T5.0. G3.3	A G11.5
55	E D8.7. G6.7. A1.5. Q1.2	E Q2.1. G1.2. A.9	E D5.1. A2.1. G1.7	E D4.1. G1.6	E	E	E	E
56	G	G	G	G	G	G	G	G
57	C	C	C	C	C	C	C	C
58	R K12.0	Q E1.8	K R17.1N.10	K R11.3	R K26.5	Q	Q	Q
59	Q K4.6	Q R2.2. K1.3	Q	Q	K	Q	Q E1.3	Q
60	I	I A1.8	I	I L4.5	I	I L10.4	I L25.4V1.3	L I41.0
61	L I5.2. M3.7	M I38.6L13.0	I M33.9L17.3	M L33.9I24.0	I	M	I L8.5. M8.5	M I16.4
62	G E17.7A4.1. R3.0. V1.9. Q1.5. I.10	E K7.7. G5.5. N2.4. I1.8. D1.8. A1.5	K R13.3Q13.0T8.5. N3.2. M2.4. S1.1	G E30.3A19.2	G A17.6	M V16.7T12.5	E	E
63	Q	Q K1.6	Q	Q A4.1. K1.4	Q	Q	Q	Q
64	L I1.0	L I3.6	L I3.4	L I4.3	L	L	L	L
65	Q H3.8	Q	Q H14.8R2.3. N2.1	Q	Q	Q	Q	Q
66	P S2.3	P S28.2A1.2	P S4.0	P S2.0	P	P	S P25.1T2.0. A1.2	S P27.9
67	S A31.2T2.0	A S4.6. T1.7	A S2.7	A S43.7	S	A S20.8	T N7.1. A6.9. S2.4. I1.5. P1.4	T A41.0
68	L I4.1	L I13.5	L I4.0. V1.5	I L14.7V7.7	L	L I12.5	L	L
69	Q K4.8. R3.2. P2.7	K R10.8N7.3. Q3.9. G1.5	Q K12.2P2.3	Q K24.7N4.1. P2.0	Q	Q K27.1	K Q2.3	R K39.3G13.1
70	T	T	T	T	T	T	T	T
71	G	G	G	G	G	G	G	G
72	S T2.0	T S6.1	T S4.2	S	S	T	S	S T8.2

Figure S1 (continue)

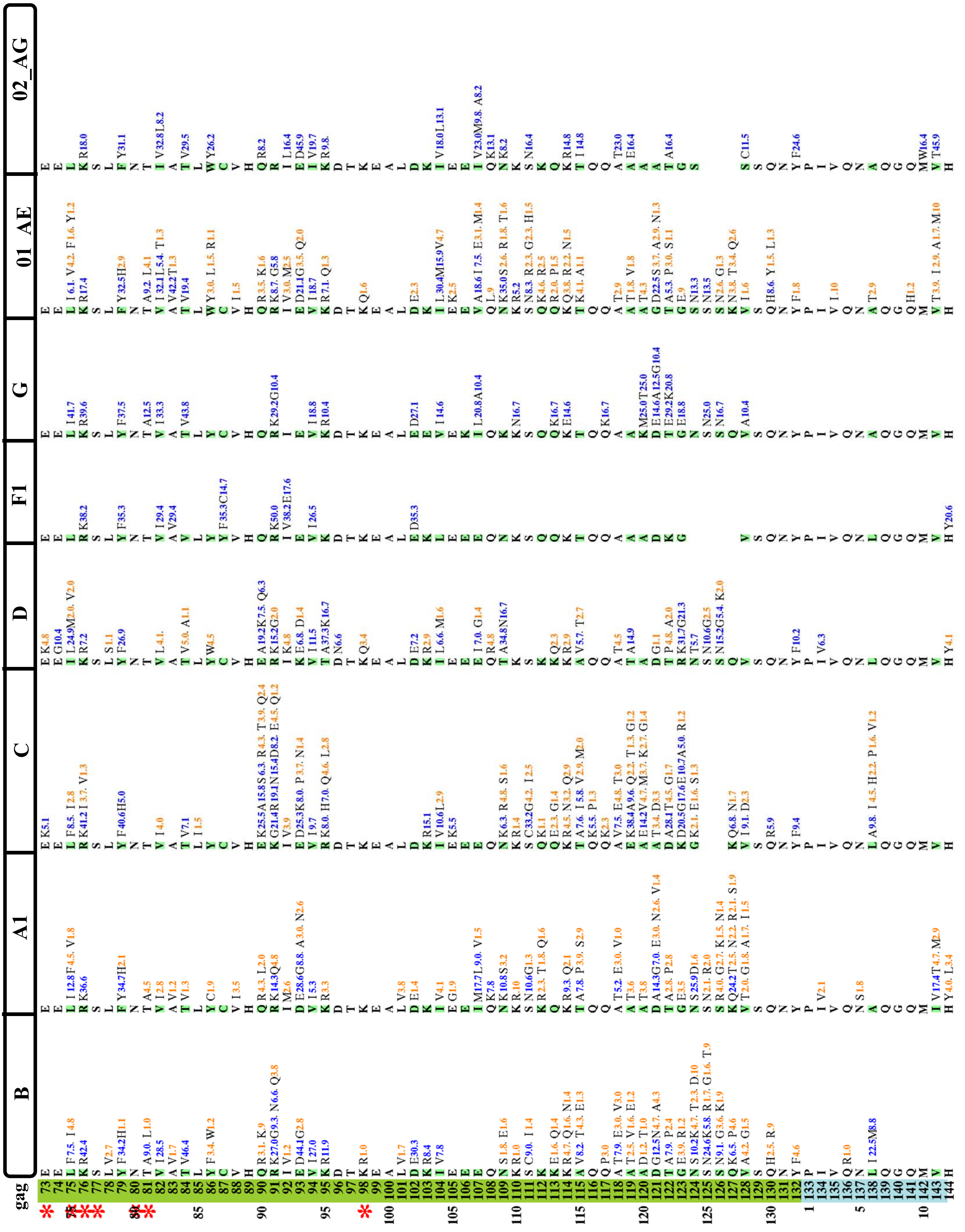

Figure S1 (continue)

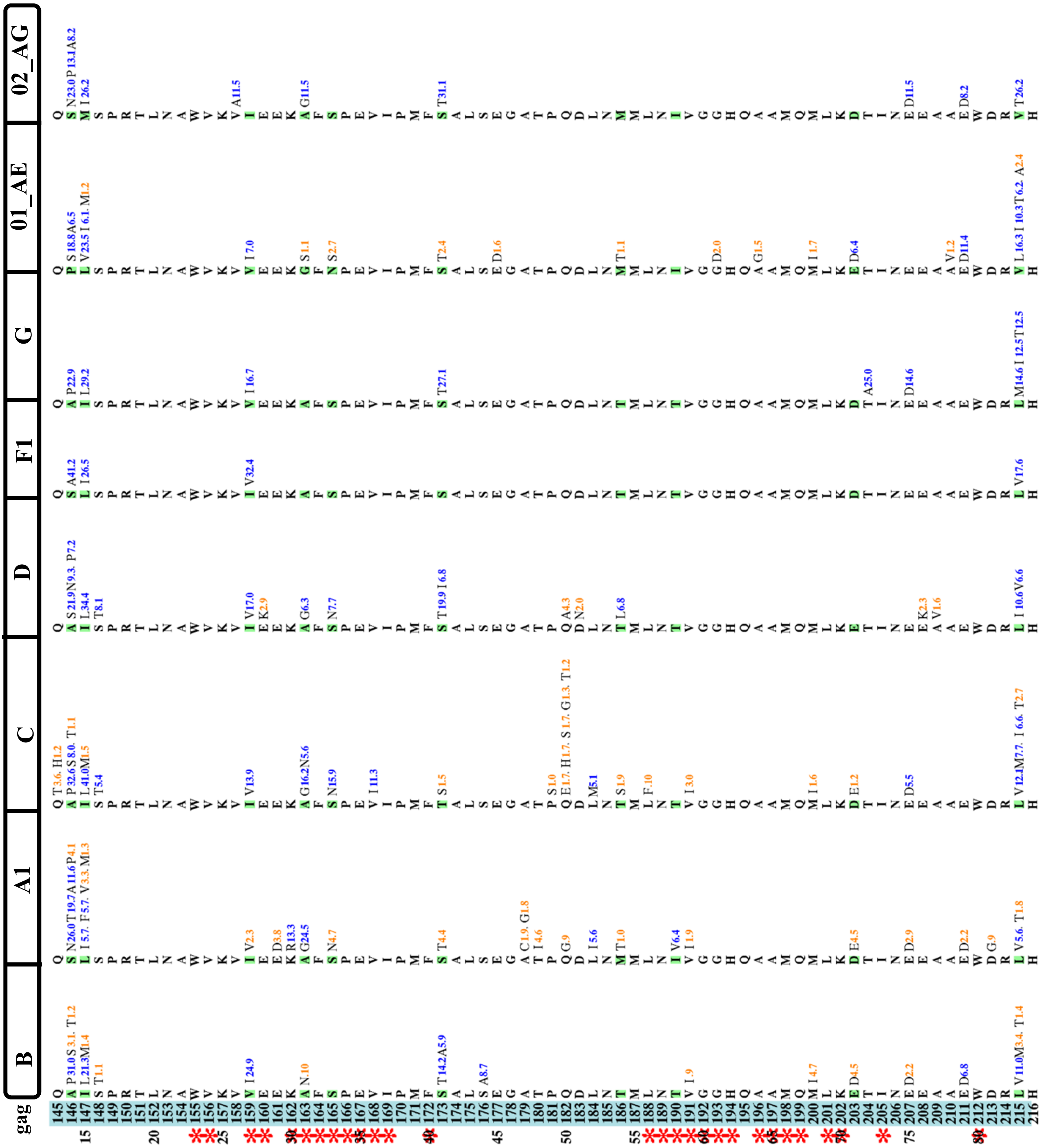

Figure S1 (continue)

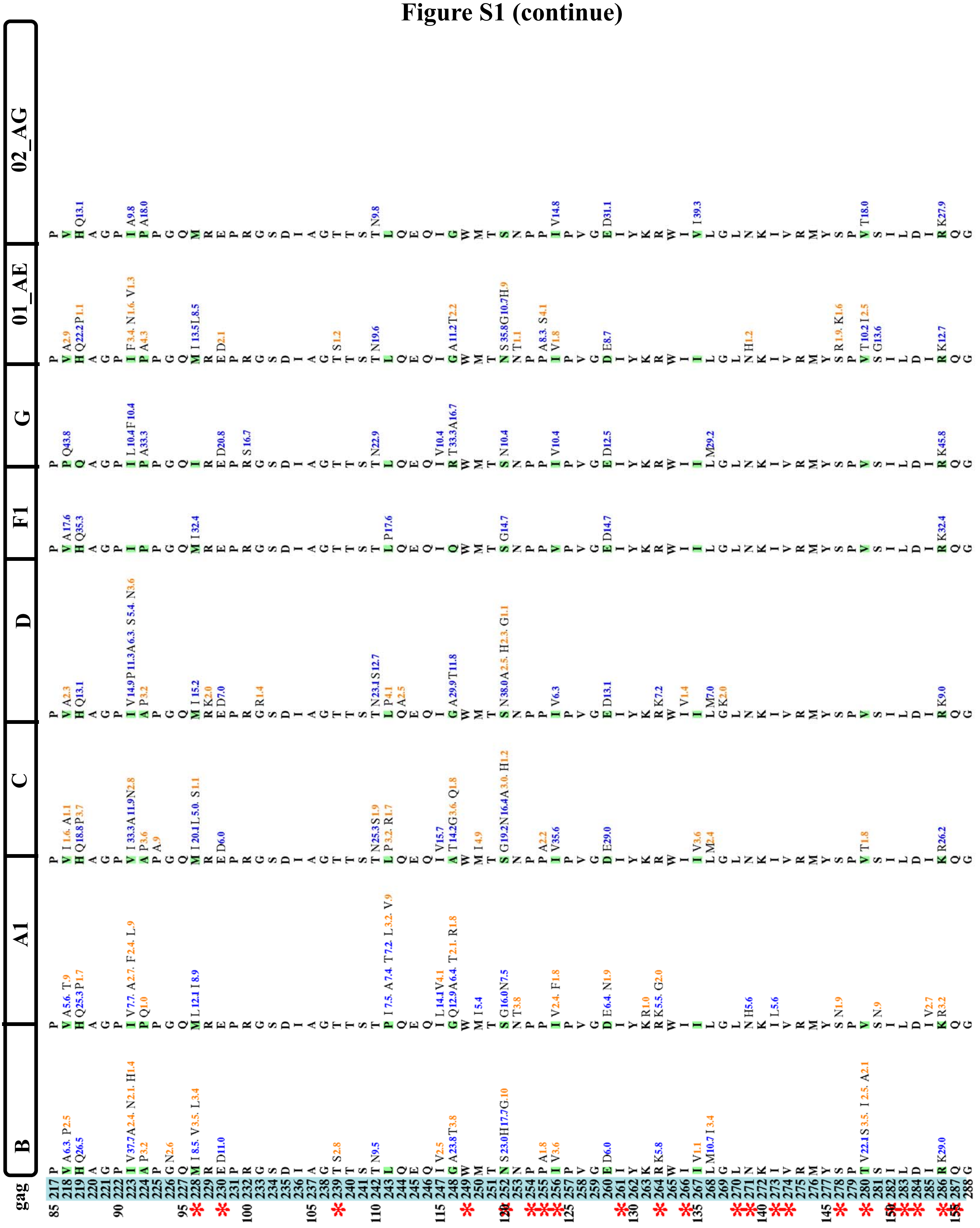

Figure S1 (continue)

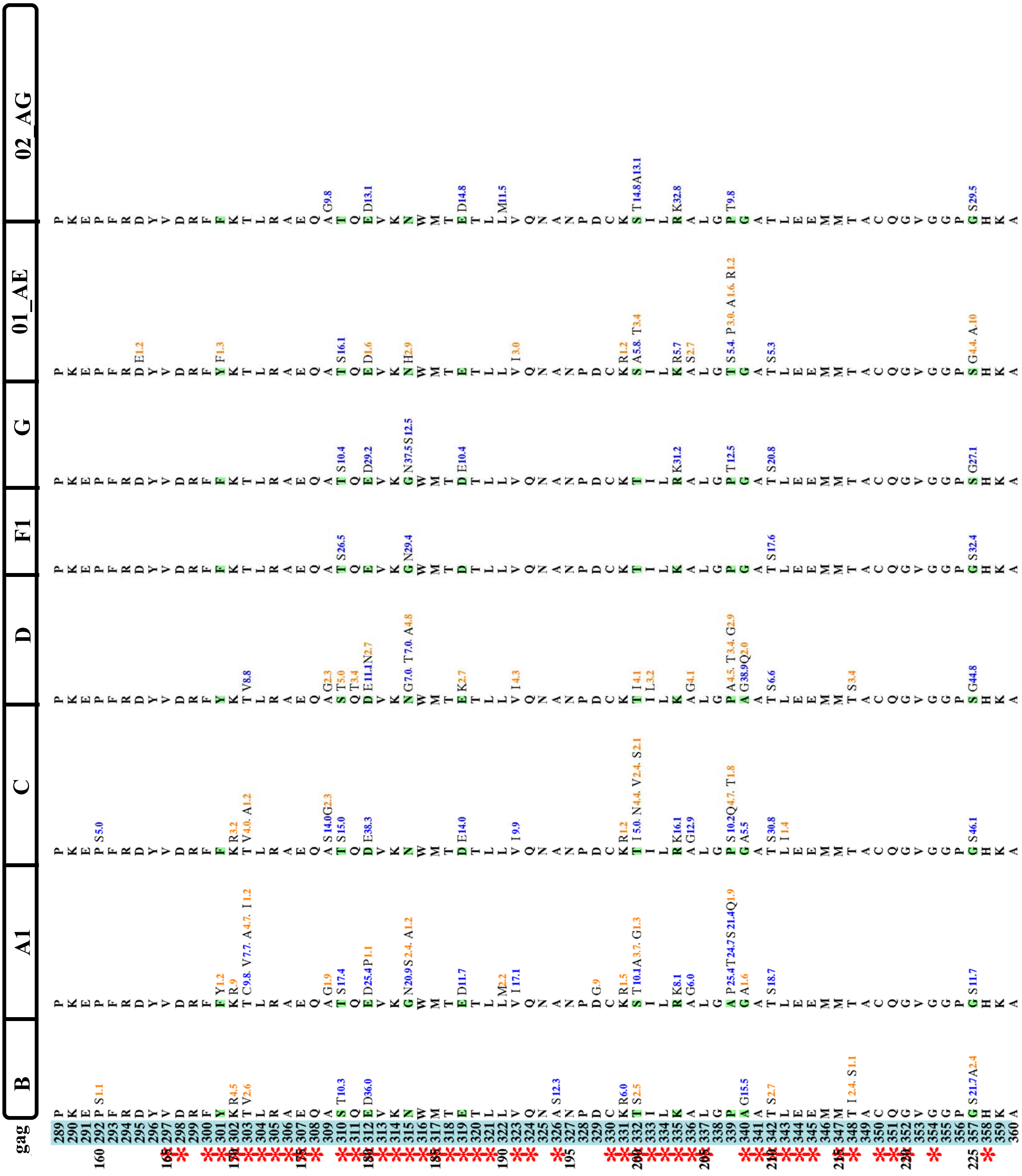

Figure S1 (continue)

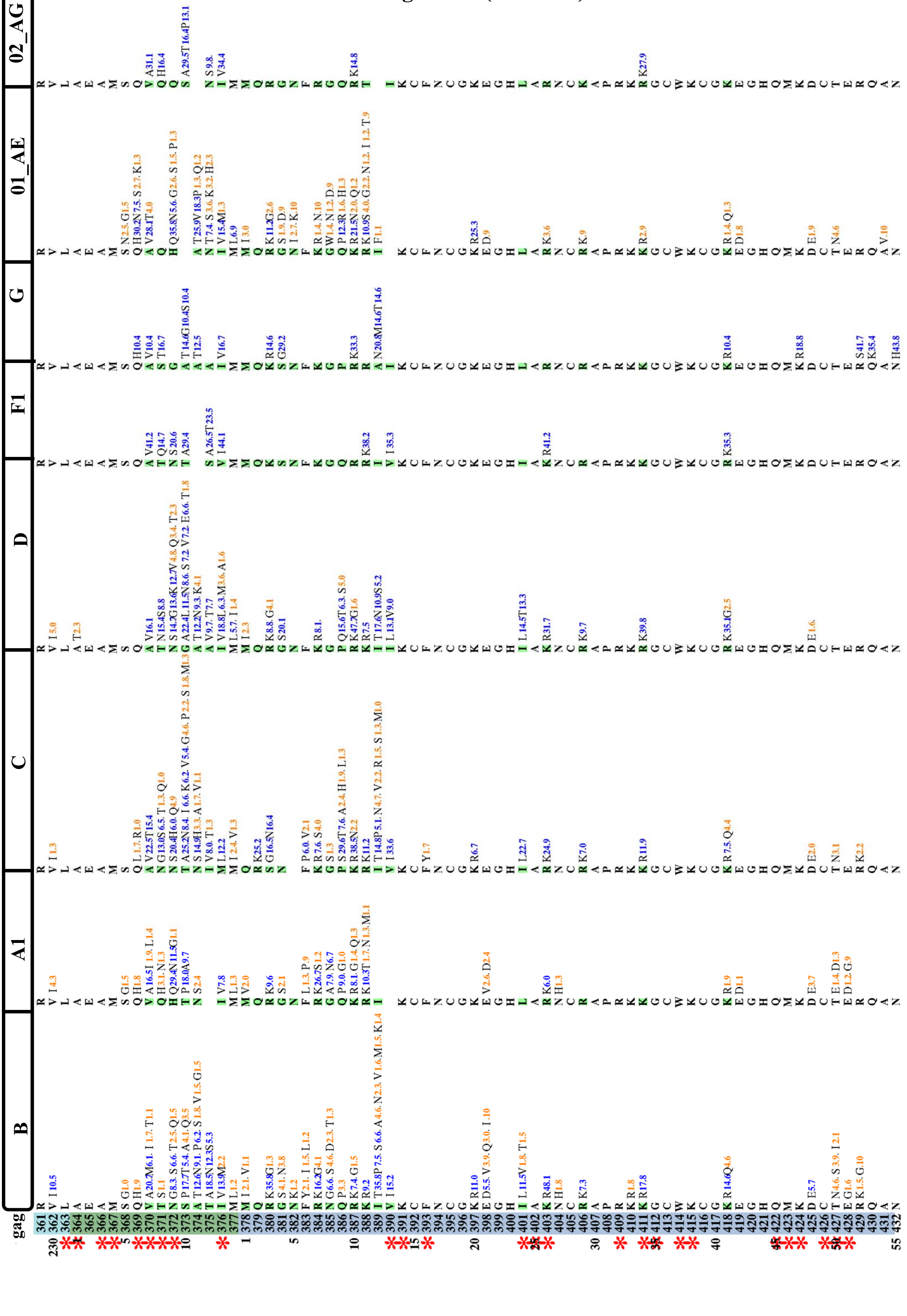

Figure S1 (continue)

	gag	B	A1	C	D	F1	G	01_AE	02_AG
1	433	F	F	F	F	F	F	F	F
	434	L	L	L	L	L	L	L	L
	435	G	G R1.0	G	G	G	G	G	G
	436	K R3.3	K R25.7	K R5.0	K R8.1	K R23.5	K R14.6	K R22.3	K R21.3
5	437	I L2.4. V1.7	I	I L2.6. F1.0. V1.0	I F14.3 L13.3	I	I	I L17.4 F4.9	I
	438	W	W	W	W	W	W	W	W
	439	P S1.4	P	P	P S7.0	P	P	P	P
	440	S	S P1.1	S	S	S	S	S P3.6	S
	441	H Y7.1. N6.0. S5.9. C2.3. Q1.5	S N16.0 H5.2. R1.5	H Q9.3. N7.8. R3.2. L2.2. Y1.8	H N9.0. R5.7. C5.2. Q4.3. Y1.4. L1.1	N H32.4	N S33.3	N H18.4 S4.8	S N29.5
10	442	K R2.3	K	K Q2.4. R2.0	K Q5.4. R1.4	K	K	K	K
	443	G E1.5	G	G	G	G	G E41.7	G	G
	444	R	R	R	R	R	R	R	R
	445	P	P	P	P	P	P	P	P
	446	G	G	G	G R1.8	G	G	G	G
15	447	N	N	N	N	N	N	N	N
	448	F	F	F	F	F	F	F	F
1	449	L P7.7	P L4.4	L I5.2. P2.7	L P1.4	L	L	P	P
	450	Q	Q	Q	Q	Q	Q	Q	Q
	451	S N16.3	N S35.0 K3.3	N S42.1 R1.2	S N14.5 R2.0	N S41.2	N S10.4	S N3.2	S
	452	R	R S1.6. K.10	R	R	R	R	R	R
5	453	P L7.3. T4.4	L P31.5 M1.0. V.10	P L11.3 T3.4. A2.8. I1.6	P T3.6	P	P T43.8	P T13.3 L5.0	P
	454	E T3.2	E	E	E	E	E	E	E
	455	P	P	P	P	P	P	P	P
	456	T S18.1	T S4.5	T S4.4	T	T	T	T S18.5	T S11.5
	457	A	A	A	A	A	A	A	A
10	458	P	P	P	P	P	P	P	P
	459	P T3.6. S1.3	P	P L1.3	P	P	P	P	P
	460	E	A	A L10.0 E4.2. V1.2	A T2.7	A	A	A	A
	461	E V2.3	E	E	E	E	E	E	E
	462	S N2.7. G2.2. I2.1	I L20.7 M4.0. S2.9. T2.6. N2.4	S Q3.9	S	S	S	N D19.5 H2.2. S1.3	S
15	463	F V2.8. L2.0	F C26.8 L10.4 S3.2. W1.7	F S3.9. A1.6. Q1.2	F L6.6	F	F L10.4	W	L F44.3
	464	R G5.2. S1.5. K1.3	G	P	G R5.7	G	G	G R6.1. E1.3	G
	465	F	M F2.6. I2.4. V1.2	R K13.2 P3.9. E3.5. G1.6. S1.4	F	F	F	M R2.9. I1.7. T1.6. V1.3	M A13.1
	466	G R1.3. V1.0	G R8.1. E4.2. A2.1	F P5.0. H2.3	G	G R23.5	G	G R6.4. E5.2	G
	467	E G1.6	E G6.6	E Q4.6. G3.1. R3.0	E	E	E	E G4.4	E
20	468	E G5.7	E G1.2	E G4.1. R3.6. A2.4. Q.10	E K2.9	E	E	E G3.8. D2.7. K1.3	E
	469	T K2.5. A2.5. I2.4	I T7.3. M4.9. V4.7	T A6.0. N2.3. P1.5. I1.4. R1.0	I V12.2 T7.2. M1.6	I T17.6	I T12.5	I T30.1 M11.2 V3.6. L3.2. R2.1. K1.8	I T18.0 V16.4
	470	T A17.0 I1.8		K T2.1	T P13.6 A12.9		A		T P16.4
	471	T A8.3. P1.9. N1.3	A T35.3 V3.5. S1.5	T P12.7 A2.8. L2.3. N1.2	P I3.4. T1.1	T		T A7.2. N3.1. I3.0. P2.5. V1.3	S P24.6
	472	P	S P9.8. F3.8	P L5.6. S5.3. H3.2	P S4.8. L2.5	P	P	S P11.2 F1.1. T1.1	S P27.9
25	473	S P33.7 A1.7. V1.5	P S4.6. H1.1	A T6.4. S3.9. P2.7. V2.2	S P16.7 A6.8	S	S A45.8	L S26.0 F4.2. P3.9. V1.9	P Q18.0
	474	Q	P L17.8 Q10.7	P L8.3. Q3.7	Q	P	P	L P21.4 Q4.7. M3.6. T1.4	K Q14.8
	475	K R5.2	K Q2.6. R2.1	K R7.4. Q1.5	K N3.2. R1.1	K	K	K R5.7	Q
	476	Q P4.6. K2.4	Q E2.6. P.10	Q R2.7. K.9	Q R1.4.	Q	Q	Q	E
	477	E G6.7. D2.9	E G3.1. Q2.6	E D1.8	E	E	E	E G1.2. R1.2	P
30	478	P T14.1 Q12.9 S5.8. L2.7. K1.1	Q K2.0	P Q24.4 S17.7 L11.1 T2.8. K1.5. M1.3	Q K9.0. P5.4	Q	P Q12.5	Q	R G29.5 K11.5
	479	I T11.5 K5.4. R4.8. V3.2. M1.3	K R18.5 T2.1. E1.3	K R11.6 G3.5. N2.3. Q1.2	K E3.2. T2.3. Q1.4.	K	K R27.1	K R2.2	D
	480	D N2.7. E1.4	D E8.4. N2.9. G1.3	D E3.9. N1.5	D E12.0	D E32.4	E K12.5	D N2.6. G1.7. E1.1	K
	481	K E6.1. R4.6. Q2.3	R K15.2 G1.4	R K8.8. G1.8	K	E	K E10.4	K R9.9. Q5.4. E3.3	G E36.1
	482	E D10.2 G4.2	E D1.1. N.10	E	E D5.0.	G	E	E D12.0 G5.3	L
35	483	L M13.5 R3.8. K2.2. Q1.9. T1.7. V1.5	Q P5.1. L4.6. R1.2	P	L	L Q35.3	L	H Q12.0 P9.2. R2.0. L1.7. Y.10	Y
	484	Y P2.1	A G1.0. V.10		Y C10.4	Y	Y P14.6	P S3.3. H1.7	P
	485	P	P	P T6.9. A2.9	P	P	P	P L2.0	P
	486	L S6.3. M2.6. V1.5	L S20.0 A1.8	L S7.5	L S9.3. V1.4	L	L	L S36.5 V1.9	L
	487	A T34.5 V1.2. S.9	V I42.6 T5.2. A1.7	T I29.4	A T24.2 V6.1. S1.4	A	A T18.8	V I13.8 A2.1. T1.9. P1.2	T A34.4
40	488	S A1.2	S	S A4.5	S A3.6	S	S	S	S
	489	L	L	L	L	L	L	L	L
	490	R K44.2	K R3.1	K R9.0	K R15.4	K	K	K	K
	491	S	S	S	S L2.0	S	S	S	S
	492	L	L	L	L	L	L	L	L
45	493	F	F	F	F	F	F	F	F
	494	G	G	G	G	G	G	G	G
	495	N S10.0	N S2.4	S N23.6	N S5.4	N	S N39.6	N S4.4	N
	496	D	D	D	D	D	D	D	D
	497	P H.9	P L24.5	P	P	P Q23.5	P	P L2.3. Q1.8	P Q23.0
50	498	S L14.9	L S9.7	L W2.3. S1.3	S L35.5			L S1.2	
	499	S L6.5	S L3.1	S L5.5	S L5.2	S		S L3.9	
	500	Q	Q	Q	Q	Q		Q	

Figure S2

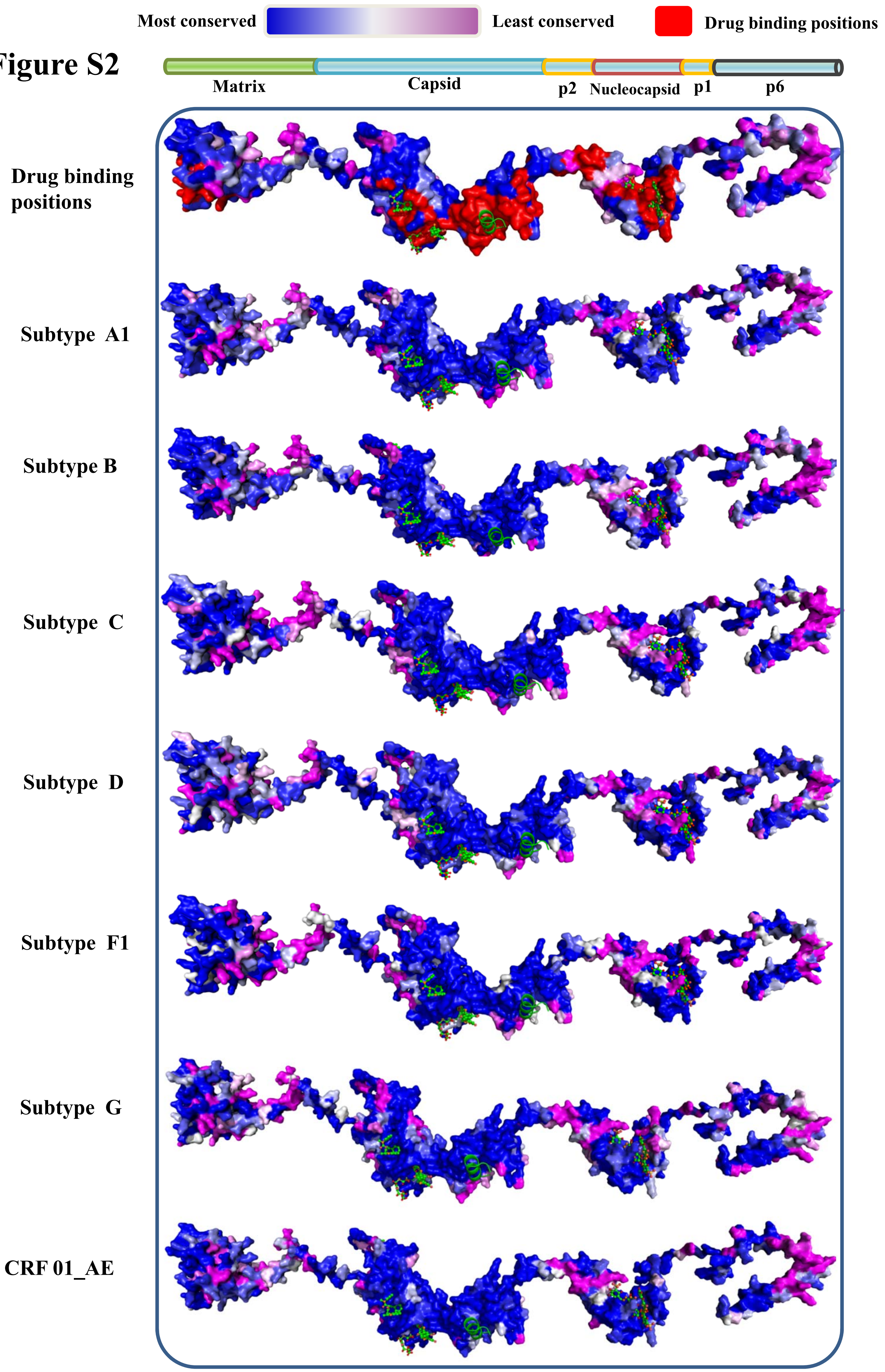
Most conserved
Least conserved
Drug binding positions
Matrix
Capsid
p2
Nucleocapsid
p1
p6
Drug binding positions
Subtype A1
Subtype B
Subtype C
Subtype D
Subtype F1
Subtype G
CRF 01_AE

Figure S2 (continue)

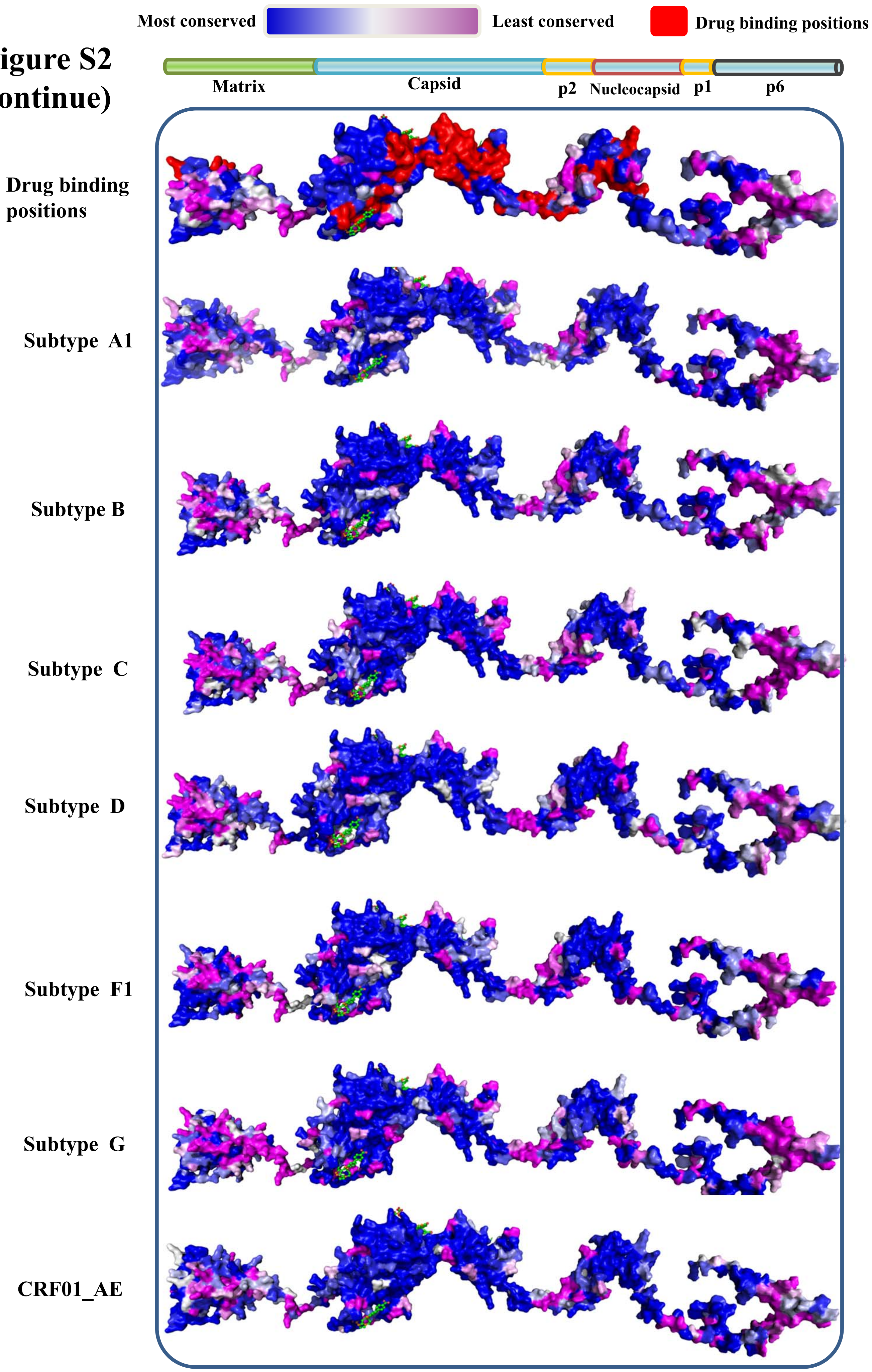

Fig S3

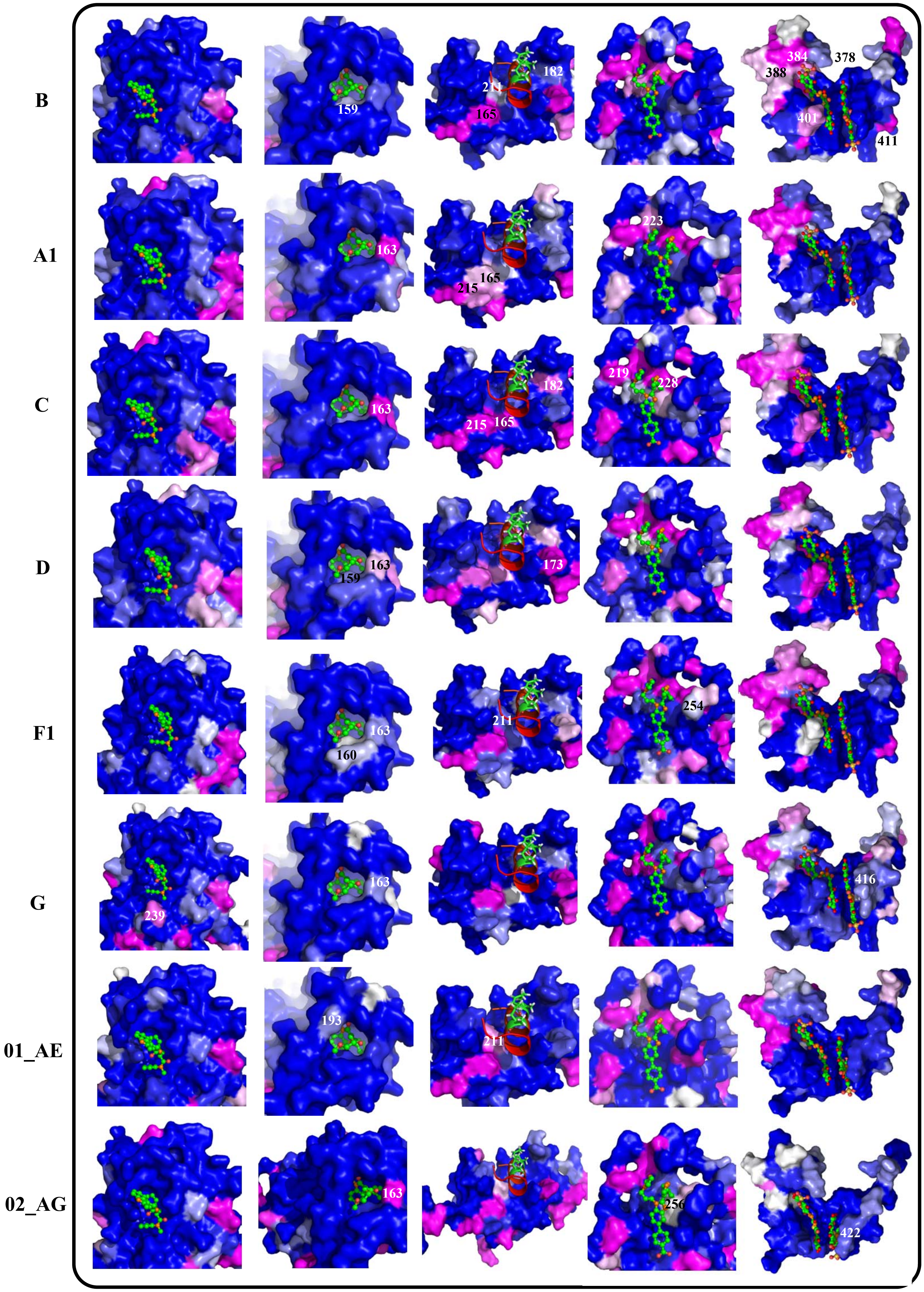

Binding pockets 1
Binding pockets 2
Binding pockets 3
Binding pockets 4
Binding pockets 5
B
A1
C
D
F1
G
01_AE
02_AG
159
211
182
165
384
378
388
401
411
163
165
215
223
163
182
215
165
219
228
163
159
173
163
160
211
254
239
163
416
193
211
163
256
422

Fig S4(A)

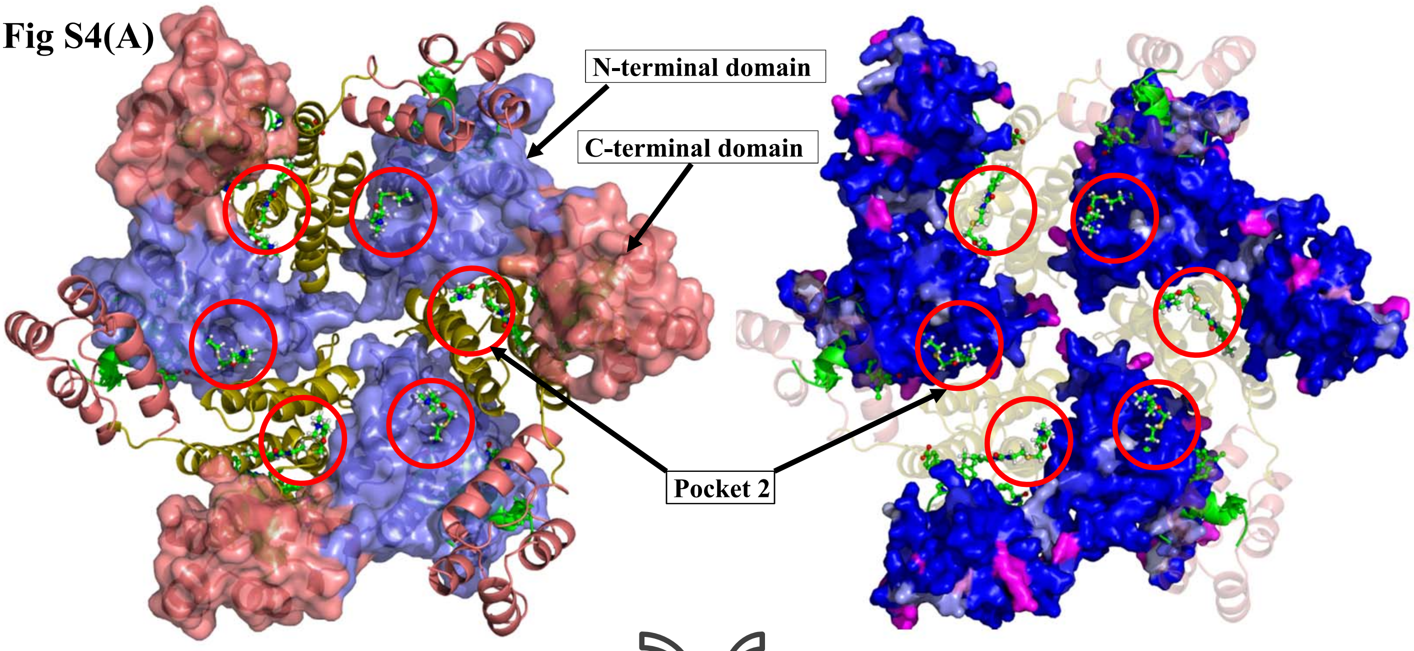

Pocket 2: NTD-NTD interaction

Fig S4(B)

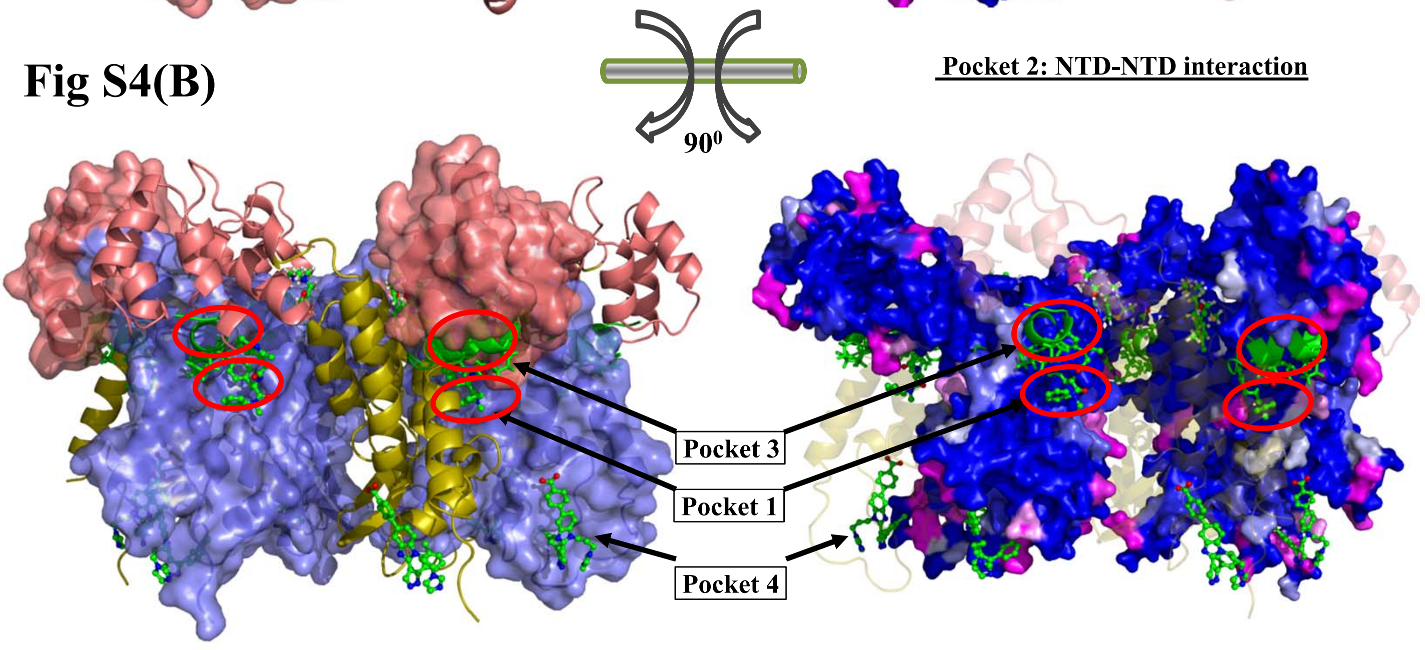

Pocket 1: NTD-CTD interaction

Pocket 3: NTD-CTD interaction

Fig S4(C)

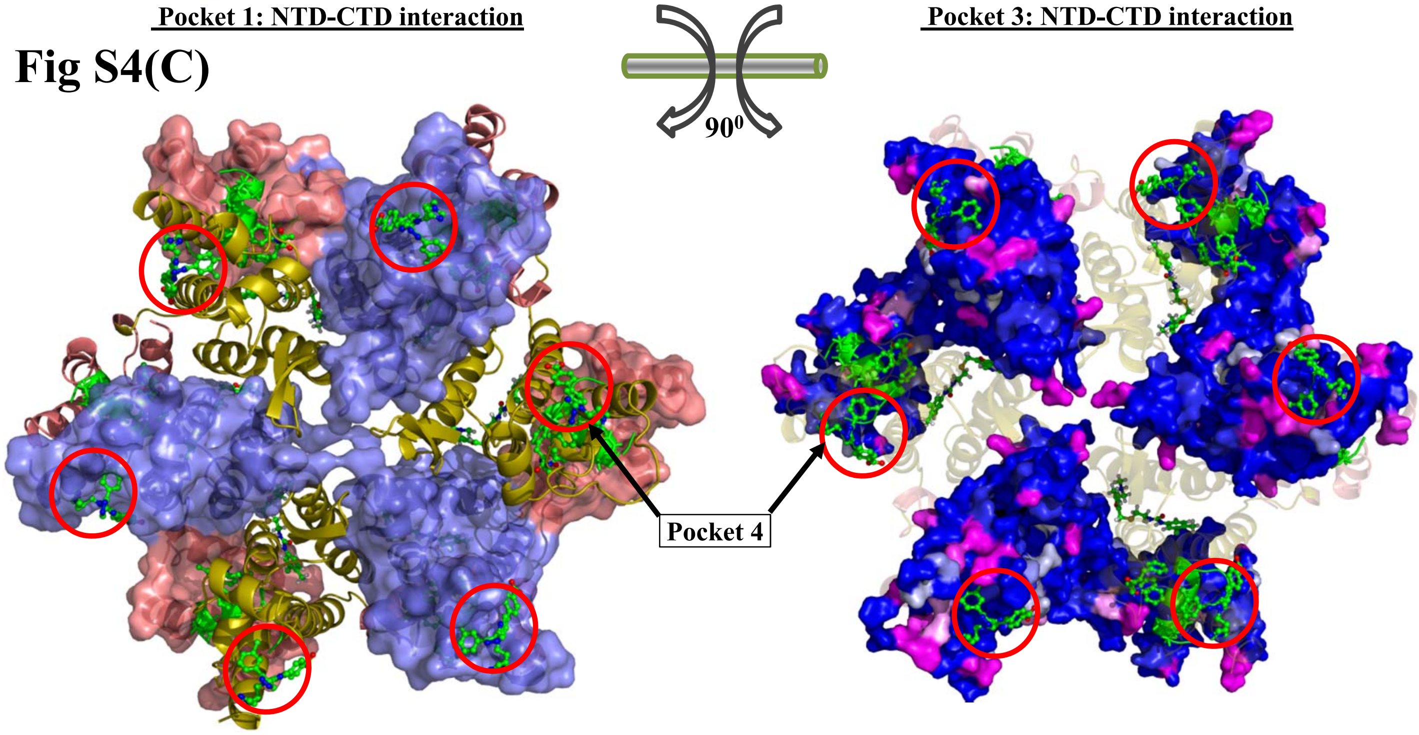

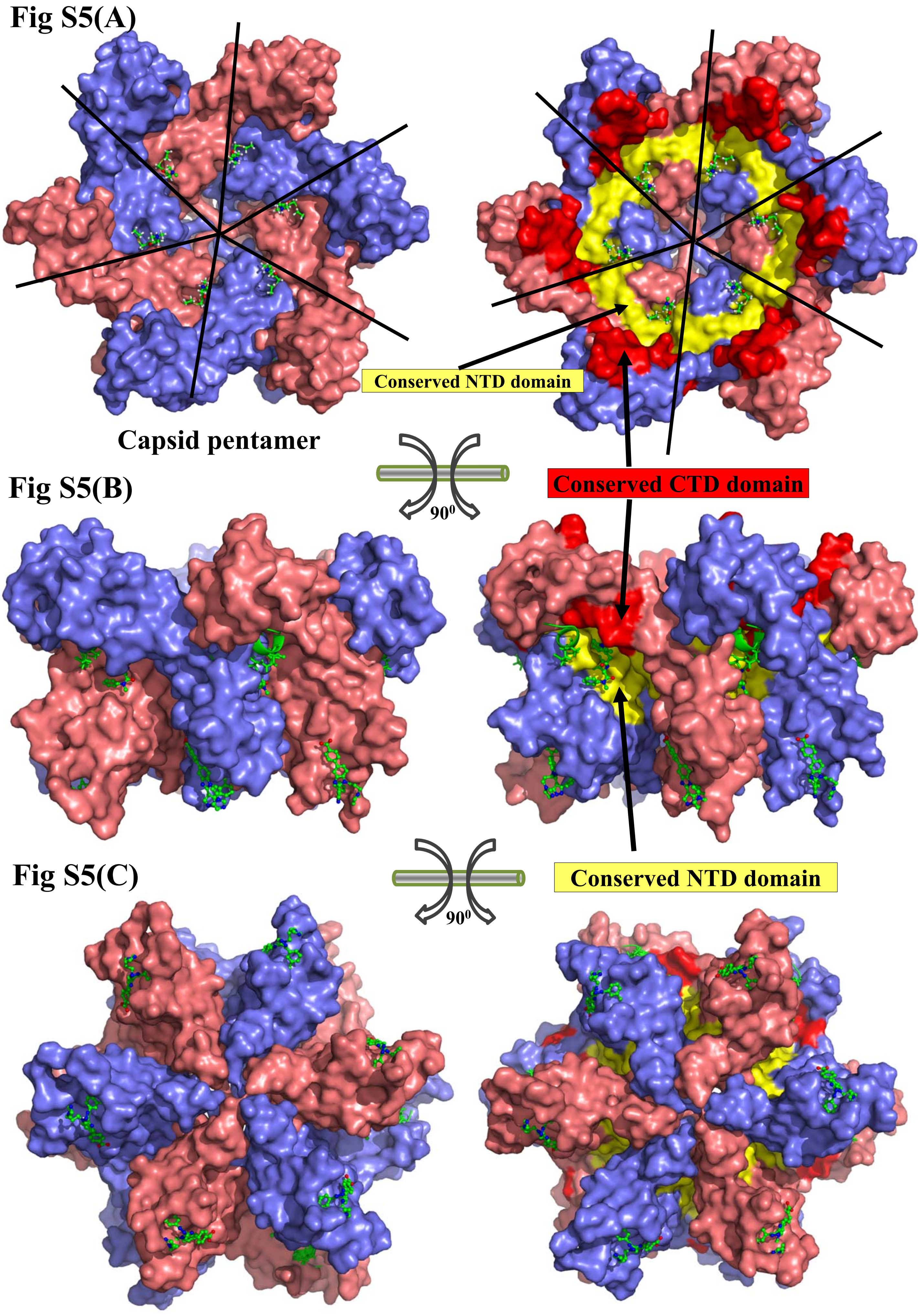
Fig S5(A)
Capsid pentamer
Conserved NTD domain
Conserved CTD domain
90⁰
Fig S5(B)
Conserved NTD domain
Fig S5(C)
90⁰

Fig S6(A)

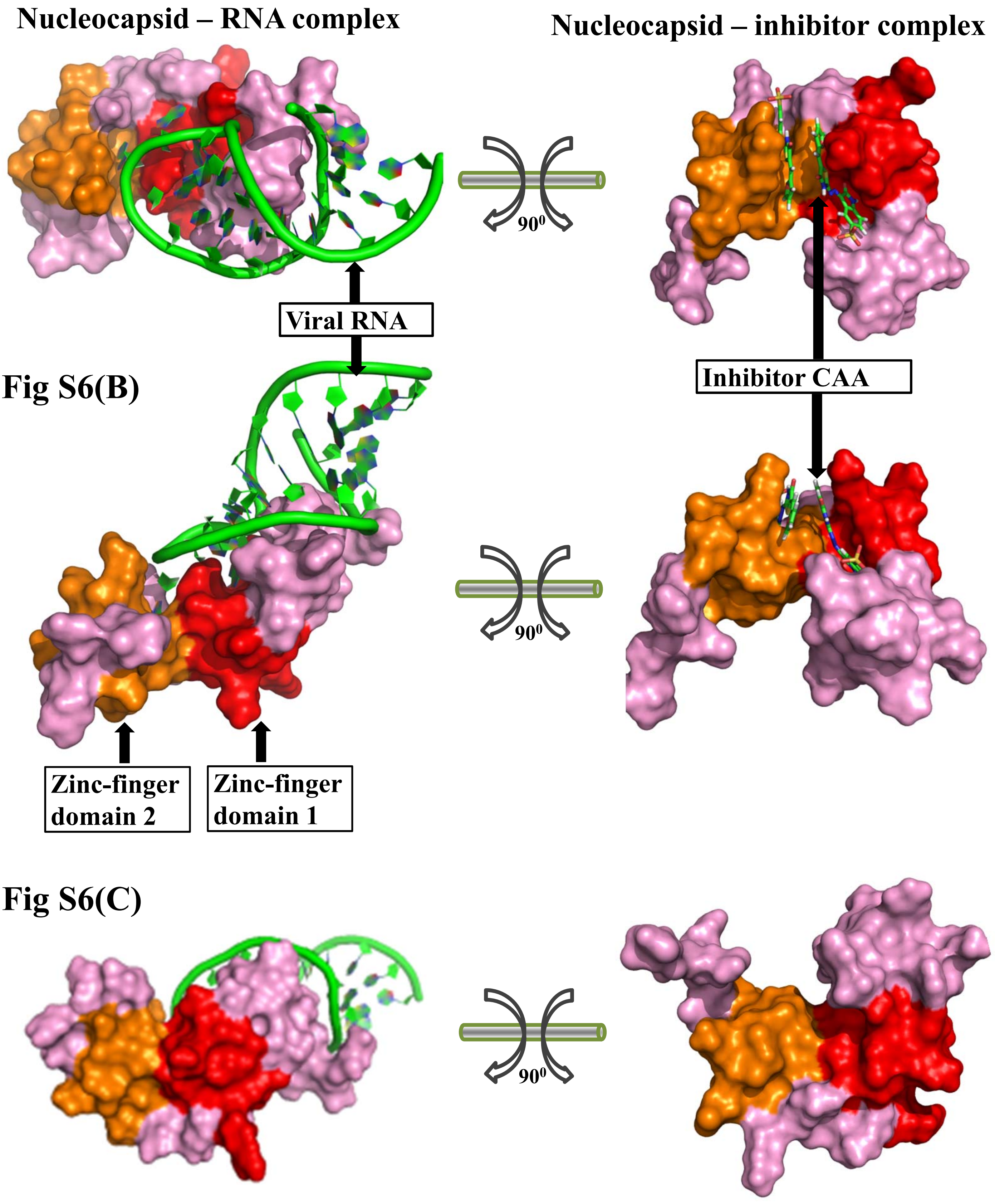
Nucleocapsid – RNA complex
Nucleocapsid – inhibitor complex
90⁰
Viral RNA
Inhibitor CAA
Fig S6(B)
90⁰
Zinc-finger domain 2
Zinc-finger domain 1
Fig S6(C)
90⁰
