# Supplementary material for: Functional conservation of HIV-1 Gag: implications for rational drug design
Source: Retrovirology. 2013 Oct 31;10:126. doi: 10.1186/1742-4690-10-126 (PMC4228425; doi:10.1186/1742-4690-10-126)
Supplement: Additional file 4 — The Matlab toolbox developed for conservation analysis, inter- and intra-subtype diversity analysis. The full-length gag sequence datasets are also included. [file 1742-4690-10-126-S4.zip › MatlabToolbox_ModelManual.pdf]

Additional file 2: Notes

Title

Functional conservation of HIV-1 gag: implications for rational drug design

Authors and Affiliations

Guangdi Li[1], Jens Verheyen[2], Soo-Yon Rhee[1,3], Arnout Voet[4], Anne-Mieke Vandamme[1,5], Kristof Theys[1]

*Corresponding author: Kristof Theys Kristof.Theys@rega.kuleuven.be

1 Rega Institute, Department of Microbiology and Immunology, KU Leuven, Leuven, Belgium

2 Institute of Virology, University hospital, University Duisburg-Essen, Essen, Germany

3 Division of Infectious Diseases, Department of Medicine, Stanford University, Stanford, California, USA

4 Zhang IRU, RIKEN Institute Laboratories, Hirosawa 2-1, Wako-shi, Saitama, Japan

5 Centro de Malária e Outras Doenças Tropicais and Unidade de Microbiologia, Instituto de Higiene e Medicina Tropical, Universidade Nova de Lisboa, Lisboa, Portugal

Email: Guangdi Li liguangdi.research@gmail.com
Jens Verheyen jens.verheyen@uk-essen.de
Soo-Yon Rhee syrhee@stanford.edu
Arnout Voet arnout.voet@fys.kuleuven.be
Anne-Mieke Vandamme annemie.vandamme@uz.kuleuven.ac.be
Kristof Theys Kristof.Theys@rega.kuleuven.be

Note S1: Mathematical model of conservation index

We analyzed the degree of positional conservation in the multiple sequence alignment (MSA), taking into account of stereochemical variability between amino acids. Adapted from the conservation analysis in Karlin and Brocchieri [1, 2], a conservation index (CI) was calculated for each position by averaging pairwise dissimilarity scores between all AAs using BLOSUM62 matrix [3].

Amino acid substitution matrices (e.g. BLOSUM62) are designed for estimating the occurrence of each possible pairwise substitution over evolutionary time. While the genetic code allows the translation of similar codons into the same synonymous or similar AAs, mutating one AA to another AA with substantially different biochemical properties can affect protein folding or activity [4]. In a substitution matrix, the nondiagonal pairwise scores how likely an AA is to be substituted by another in a homologous protein and the diagonal scores indicate how likely one AA is to be substituted at all [5]. For instance, a negatively charged residue like aspartic acid D is more likely to be replaced by the other negatively charged residue glutamic acid E, than it is to be mutated into positively charged histidine H. In BLOSUM62 matrix, D to E is scored 2, while D to H is -1.

Adapted from Karlin and Brocchieri [1, 2], conservation index (CI) of position x is calculated as:

$$CI(x) = 1 - \frac{2}{N(N-1)} \sum_{i=1}^{N} \sum_{j=i+1}^{N} \frac{S(x_i, x_j)}{\sqrt{S(x_i, x_i) S(x_j, x_j)}}$$

Where x_i is the amino acid form at the position x of the ith sequence in the MSA, N is the number of sequences in MSA, $S(x_i, x_j)$ is the similarity score between amino acid form x_i and x_j. Suggested in Karlin and Brocchieri [1, 2], we adapted the similarity matrix BLOSUM62 to provide the similarity scores for $S(x_i, x_j)$. Since denominators should not be zero, the values of BLOSUM62 M are linearly transformed into positive by adding the absolute value of minimum score $|\min(M)| + 1$. In our analysis, the conservation index of positions with less than 20% gaps is calculated, and the amino acid comparisons were restricted to 20 amino acids (e.g. ARNDCQEGHILKMFPSTWYV). Note that if no natural variations exist at conserved position x, then $CI(x) = 0$ otherwise, $0 < CI(x) < 1$. Given BLOSUM62 as the similarity matrix for $S(x_i, x_j)$, it can be shown that $0 \le CI(x) \le 0.9278$. Besides, the relationship between conservation index and pairwise diversity can be described by the Proposition 1, which explains that conservation index is equal to or less than pairwise diversity. Note that pairwise diversity is defined as: $\frac{2}{N(N-1)} \sum_{i=1}^{N} \sum_{j=i+1}^{N} \delta(x_i \ne x_j)$, where δ denote the Kronecker symbol, $\delta(x_i = x_j) = 1$ if x_i is equal to x_j; otherwise 0.

Proposition 1. Suppose x is a position in MSA and x_i is a polymorphism at x, $P(x_i)$ is the prevalence of x_i, let $CI(x)$ and $Diversity(x)$ denote conservation index and pairwise diversity, respectively, then:

$$CI(x) \le Diversity(x)$$

Proof: Assume that an amino acid similarity matrix S (e.g. BLOSUM62) satisfies $S(x_i, x_i) \ge S(x_i, x_j)$. We have:

$$\delta(x_i \ne x_j) \ge 1 - \frac{S(x_i, x_j)}{\sqrt{S(x_i, x_i) S(x_j, x_j)}}$$

It can then be concluded that:

$$\begin{aligned} CI(x) &= 1 - \frac{2}{N(N-1)} \sum_{i=1}^{N} \sum_{j=i+1}^{N} \frac{S(x_i, x_j)}{\sqrt{S(x_i, x_i) S(x_j, x_j)}} \\ &= \frac{2}{N(N-1)} \sum_{i=1}^{N} \sum_{j=i+1}^{N} [1 - \frac{S(x_i, x_j)}{\sqrt{S(x_i, x_i) S(x_j, x_j)}}] \\ &\le \frac{2}{N(N-1)} \sum_{i=1}^{N} \sum_{j=i+1}^{N} \delta(x_i \ne x_j) \\ &= Diversity(x) \end{aligned}$$

□

The above section explained relationship between CI(x) boundary and pairwise diversity; we now discuss the properties of the relationship between CI(x) and accumulative polymorphism prevalence. Let $C(x)$ be the cumulative polymorphism prevalence at position x, a trivial observation can be found as: $CI(x) \le Diversity(x) \le \min\,[1, \frac{2N}{N-1}(C(x)+C^2(x))]$. This is derived from the following equations that $Diversity(x) = \frac{2N}{N-1}\sum_a \sum_{b\neq a} P(x=a)P(x=b)$ and $C(x) = \sum_{P(x=a)\le 0.5} P(x=a)$, where a and b are two amino acid forms at position x in MSA.

Theoretical results did not yield a precise value for the upper boundary of CI(X) using C(X). We therefore used our HIV-1 gag datasets to show the relationship between $CI(x)$ and cumulative polymorphism prevalence regarding the identification of conserved positions. Given the cutoff 0.01 for both $CI(x)$ and cumulative polymorphism prevalence, we compared the results from both measurements. Suppose S_1 is the set of conserved positions given the cutoff of $CI(x)$, S_2 is the set of positions with cumulative polymorphism prevalence less than 0.01. We found that only 5 out of 147 positions in S_1 were different from S_2, and 6 out of 149 positions in S_2 were different from S_1. The two measurements reach up to 95.9% (6/149) common predictions. In other words, using $CI(x)$ tests to identify conserved sites at cutoff 0.01 can approximately guarantee cumulative polymorphism prevalence below 0.01.

Herein, we provide an adapted example from Valdar [5] to compare conservation index with other state-of-the-art conservation methods (i.e. Shannon entropy, Jensen-Shannon diversity, relative entropy, property relative entropy, sum of pairs [6]). We used our Matlab package to calculate Shannon entropy and the python software from Capra and Singh [6] to calculate the other measurements (default settings).

Table S1. Comparison of conservation methods given a simple sequence example.

Example	Pos1	Pos2	Pos3	Pos4	Pos5	Pos6	Pos7	Pos8	Pos9
Seq1	E	D	D	D	D	D	I	P	D
Seq2	E	D	D	D	D	D	I	P	V
Seq3	E	D	D	D	D	D	I	P	Y
Seq4	E	D	D	D	D	D	I	P	A
Seq5	E	D	D	D	D	D	L	W	T
Seq6	E	D	D	D	E	E	L	W	K
Seq7	E	D	D	D	E	E	L	W	P
Seq8	E	D	D	D	E	E	L	W	C
Seq9	E	D	D	D	E	F	V	S	R
Seq10	E	D	E	F	F	F	V	S	H
Methods									
Conservation index	0	0	0.0665	0.1636	0.3107	0.4006	0.1580	0.5874	0.6730
Shannon Entropy	0	0	0.1412	0.1412	0.4097	0.4472	0.4581	0.4581	1
Property entropy	0	0	0.0418	0.1253	0.1896	0.1703	0.1998	0.4889	0.6355
Jensen-Shannon	0.8367	0.8299	0.8007	0.7621	0.7102	0.6567	0.6507	0.6075	0.5497
Relative Entropy	0.9447	0.9481	0.9048	0.8257	0.7117	0.6143	0.6238	0.6070	0.5363

Property relative entropy	3.1713	3.1713	3.0608	2.8399	2.6729	2.4370	2.2060	1.7668	1.1429
Sum of pairs	5.0000	5.5500	5.1500	3.9722	2.5444	1.9166	1.7277	1.4666	-1.4888

Given the above example with 10 sequences (Seq1 to Seq10), the following order ranks the positions (Pos1 to Pos9) from the most conserved to the least conserved: Pos1 = Pos2 > Pos3 > Pos4 > Pos5 > Pos6, and Pos7 > Pos8 > Pos9. The most conserved positions are Pos1 and Pos2 where there is no mutation. AA change from D to E is more tolerable than from D to F, thus Pos3 is more conserved than Pos4. Pos4, with fewer mutations, is more conserved than Pos5. Pos7 which possesses all hydrophobic I, L and V are more conserved than Pos8 containing P, W, S from different AA groups (aromatic side group, hydrophobic group, polar uncharged side group). Pos9 is the most variable position with all different AAs. Table S1 also shows that conservation index at Pos4 is higher than at Pos7, but Pos4 has fewer mutations than Pos7. This is due to the fact that all three amino acids at Pos7 (valine, isoleucine and leucine) have similar structures and belong to the same function group (http://en.wikipedia.org/wiki/Amino_acid). BLOSUM62 assigns high positive scores to the replacement of valine by isoleucine (s = 3) and by leucine (s = 1). Although Pos4 has only one mutation, the substitution D to F represents an AA replacement across different functional groups. Aspartic acid D from the carboxylic acid group is negatively charged without a benzene ring, while phenylalanine F from the hydrophobic group has a large benzene ring with no charges, resulting in a low substitution score of -3 between D and F in BLOSUM62. This example demonstrates how the conservation index takes into account biochemical differences between amino acids as well as the prevalence of AA substitutions.

We found that CI was a robust estimation of the conserved sites for three reasons: (1) positions with no natural variations in the MSA have equal CIs. This is not the case with Jensen-Shannon diversity score, for instance. (2) Positions with higher natural variations have higher CIs. This is not the case with property entropy, for instance. (3) The biochemical similarities between amino acids are taken into account. This is not the case with Shannon entropy where all amino acids are treated equally. Regarding the difference between state-of-the art methods, it has been described extensively in [5] [6]. Given 4130 full-length HIV-1 subtype B gag sequences, Figure S1 demonstrates the distribution of conservation scores in HIV-1 subtype B gag using conservation index, Shannon entropy and relative entropy. Figure S2 demonstrates the comparison of Shannon entropy and conservation index using full-length protease sequences sampled from 723 HIV-1 subtype B patients, downloaded from HIV Los Alamos Database.

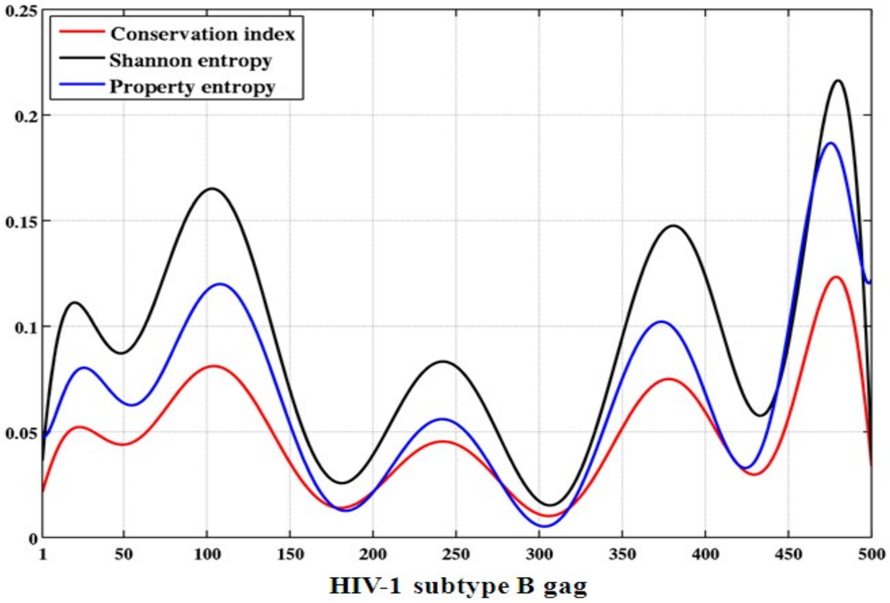

Figure S1. Amino acid conservation in HIV-1 full-length gag analyzed by conservation index, Shannon entropy and relative entropy.

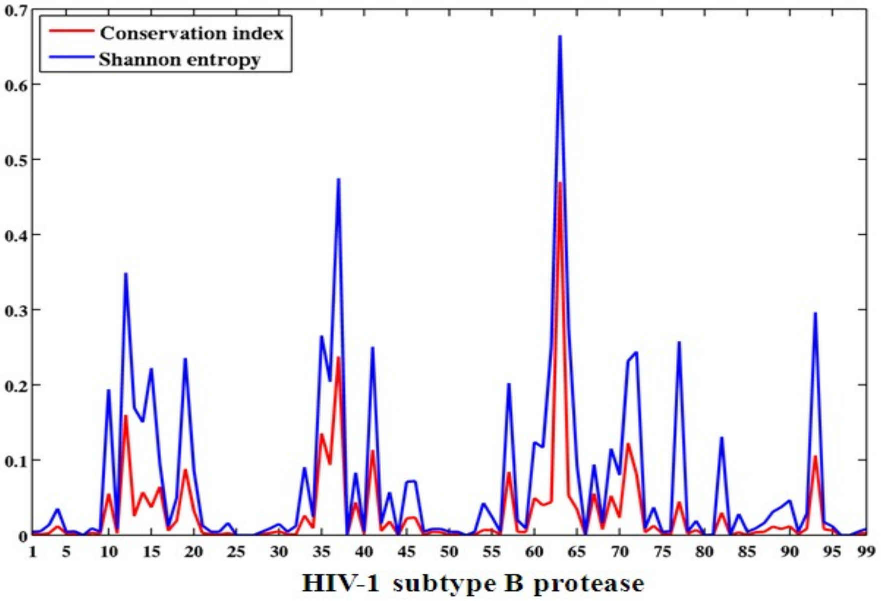

Figure S2. HIV-1 protease conservation analyzed by conservation index and Shannon entropy.

The above two figures demonstrate that the three methods show similar patterns in full-length gag conservation analysis, indicating that conservation index may characterize AA conservation and yield similar patterns to entropy measurements. Note that positional conservation methods based on substitution matrices were criticized for not accounting for gaps [5], gaps were treated as missing data in our analysis and only positions with less than 20% gaps were analyzed. Regarding the performance, it is possible that other state-of-the-art methods provide equally ideal estimations of positional conservation by taking into account stereochemical sensitivity, reviewed in [5]. Taken together, our data show that conservation index provides sufficient statistical power to quantify positional conservation using the BLOSUM substitution matrix. Our Matlab toolbox and datasets are available in Additional file 4.

Note S2: Inter- and intra-subtype diversity

The amino acid inter- and intra-subtype diversity was calculated by pairwise amino acid comparisons [7]. Herein we describe the mathematical models. Suppose D is a multiple sequence alignment containing N amino acid sequences, L is the number of positions in D. Intra-subtype diversity $Diversity^{Intra}(D)$ for dataset D is calculated as:

$$Diversity^{Intra}(D) = \frac{2}{N(N-1)} \sum_{i=1}^{N} \sum_{j=i+1}^{N} \frac{1}{L} \sum_{t=1}^{L} \delta(D_i^t = D_j^t)$$

Where D_i^t is the tth amino acid form of the sequence i in dataset D, δ denotes the Kronecker symbol, $\delta(D_i^t = D_j^t)$ equals 1 if $D_i^t = D_j^t$ is true; otherwise 0.

Similarly, we can calculate the inter-subtype diversity between two sequence datasets. Suppose $D1$ and $D2$ are the multiple sequence alignments from two subtypes (e.g. subtype B and subtype C). Both have the number of sequences, N and M, respectively. The inter-subtype diversity between two subtypes $Diversity^{Inter}(D1, D2)$ is defined as:

$$Diversity^{Inter}(D1, D2) = \frac{1}{N \times M} \sum_{i=1}^{N} \sum_{j=1}^{M} \frac{1}{L} \sum_{t=1}^{L} \delta(D1_i^t = D2_j^t)$$

In our analysis, we calculated the pairwise diversity at positions on sequences with less than 20% gaps and gaps were treated as missing data. To solve the heavy computation of large sequence datasets (1000 sequences lead to half a million pairwise calculations), we implemented parallel computation with optimized memory strategy. The Matlab toolbox is available in Additional file 4.

Reference

1. Brocchieri L, Karlin S: **Conservation among HSP60 sequences in relation to structure, function, and evolution.** *Protein Sci* 2000, **9:**476-486.
2. Karlin S, Brocchieri L: **Evolutionary conservation of RecA genes in relation to protein structure and function.** *J Bacteriol* 1996, **178:**1881-1894.
3. Henikoff S, Henikoff JG: **Amino acid substitution matrices from protein blocks.** *Proc Natl Acad Sci U S A* 1992, **89:**10915-10919.
4. Gong S, Blundell TL: **Discarding functional residues from the substitution table improves predictions of active sites within three-dimensional structures.** *PLoS Comput Biol* 2008, **4:**e1000179.
5. Valdar WS: **Scoring residue conservation.** *Proteins* 2002, **48:**227-241.
6. Capra JA, Singh M: **Predicting functionally important residues from sequence conservation.** *Bioinformatics* 2007, **23:**1875-1882.
7. Rhee SY, Liu TF, Kiuchi M, Zioni R, Gifford RJ, Holmes SP, Shafer RW: **Natural variation of HIV-1 group M integrase: implications for a new class of antiretroviral inhibitors.** *Retrovirology* 2008, **5:**74.
